# Time Course of Gene Expression Profiling in the Liver of Experimental Mice Infected with *Echinococcus multilocularis*

**DOI:** 10.1371/journal.pone.0014557

**Published:** 2011-01-19

**Authors:** Renyong Lin, Guodong Lü, Junhua Wang, Chuanshan Zhang, Wenjuan Xie, Xiaomei Lu, Georges Mantion, Hélène Martin, Lysiane Richert, Dominique A. Vuitton, Hao Wen

**Affiliations:** 1 Xinjiang Key Laboratory of Echinococcosis and Medical Research Center, First Affiliated Hospital of Xinjiang Medical University, Urumqi, China; 2 Laboratoire de Toxicologie Cellulaire, EA 4267, Faculté de Médecine et Pharmacie, University of Franche-Comté, Besançon, France; 3 World Health Organization-Collaborating Centre for the Prevention and Treatment of Human Echinococcosis, Department of Digestive Surgery of Jean Minjoz Hospital, University of Franche-Comté and University Hospital, Besançon, France; Georgia Institute of Technology, United States of America

## Abstract

**Background:**

Alveolar echinococcosis (AE) is a severe chronic parasitic disease which behaves like a slow-growing liver cancer. Clinical observations suggest that the parasite, *Echinococcus multilocularis* (*E. multilocularis*) influences liver homeostasis and hepatic cell metabolism. However, this has never been analyzed during the time course of infection in the common model of secondary echinococcosis in experimental mice.

**Methodology/Principal Findings:**

Gene expression profiles were assessed using DNA microarray analysis, 1, 2, 3 and 6 months after injection of E. multilocularis metacestode in the liver of susceptible mice. Data were collected at different time points to monitor the dynamic behavior of gene expression. 557 differentially expressed genes were identified at one or more time points, including 351 up-regulated and 228 down-regulated genes. Time-course analysis indicated, at the initial stage of E. multilocularis infection (month 1–2), that most of up-regulated pathways were related to immune processes and cell trafficking such as chemokine-, mitogen-activated protein kinase (MAPK) signaling, and down-regulated pathways were related to xenobiotic metabolism; at the middle stage (month 3), MAPK signaling pathway was maintained and peroxisome proliferator-activated receptor (PPAR) signaling pathway emerged; at the late stage (month 6), most of up-regulated pathways were related to PPAR signaling pathway, complement and coagulation cascades, while down-regulated pathways were related to metabolism of xenobiotics by cytochrome P450. Quantitative RT-PCR analysis of a random selection of 19 genes confirmed the reliability of the microarray data. Immunohistochemistry analysis showed that proliferating cell nuclear antigen (PCNA) was increased in the liver of E. multilocularis infected mice from 2 months to 6 months.

**Conclusions:**

*E. multilocularis* metacestode definitely exerts a deep influence on liver homeostasis, by modifying a number of gene expression and metabolic pathways. It especially promotes hepatic cell proliferation, as evidenced by the increased PCNA constantly found in all the experimental time-points we studied and by an increased gene expression of key metabolic pathways.

## Introduction

Pathogen-induced hepatic injury has been extensively studied in animal models, and the changes in biological pathways in association with pathological progress in the liver under virus-induced infectious/inflammatory conditions have been well documented [1]–[5]. Very little is known on the capacity of helminth parasites to influence liver cell homeostasis metabolic pathways. Actually, a few helminth parasites do affect the liver [6]. Among them, infection with *Echinococcus (E.) multilocularis* larva (metacestode) affects primarily the liver and causes alveolar echinococcosis (AE) in intermediate hosts. It is an aggressive chronic parasitic infection which is characterized by an initially localized, tumor-like, multivesicular structure surrounded by an extensive fibro-inflammatory host reaction [7]. It has long been known that the liver is the key organ in *E. multilocularis* infection [7]–[11]. In humans, who behave as accidental intermediate hosts, the severity of this life-threatening disease results from both a continuous asexual proliferation of the metacestode and an intense granulomatous infiltration around the parasite; the lesions behave like a slow-growing liver cancer. *E. multilocularis* infection induces numerous pathways of the immune response in the periparasitic granuloma, at the border of the hepatic parenchyma [12], but direct consequence of the parasitic ‘tumor’ on hepatic cells and liver homeostasis has long been ignored. However, hepatomegaly is a usual symptom of AE; it has been ascribed to the liver regeneration which accompanies the pseudo-tumoral process. Only gross changes in carbohydrate metabolism [13] and in protein/albumin secretion by the liver [14], have been reported, and we recently showed that parasitic components also influenced cell signaling in hepatocytes, and especially the mitogen-activated protein kinase (MAPK) system [15].

Rather than the traditional approach of focusing on a limited number of genes at a time, cDNA microarray technology allows for a global perspective to be achieved. Many studies using microarray technologies to characterize gene expression profiles in animals exposed to pathogens have been undertaken recently [16], [17]. There are thousands of genes that have shown changes in their expression in response to pathogenic insults. However, unlike in other forms of liver injury, e.g. from neoplasms, viral hepatitis or physical injury in which gene expression profiles have already been extensively investigated [16], [18]–[20], the systemic and comprehensive analysis of gene expression during the course of the liver injury after helminth infections and especially AE is only at its beginning. The global change in gene expression in the liver of experimental mice after infection by *E. multilocularis* has just been published [21]. Gene expression was studied at 1 month after oral infection by *E. multilocularis* oncospheres, i.e. at the beginning of the chronic stage of the disease in the ‘primary infection’ model. However, we do not know if the described changes apply similarly to ‘secondary infection’, i.e. infection obtained using intraperitoneal or intrahepatic injection of metacestode. ‘Secondary infection’ is a model commonly used to study host-parasite interactions in AE because of its easier availability/safety to most of research laboratories and the possibility it offers to study the liver parenchyma distant from the lesions, a situation which mimics the disease in humans. In addition, numerous changes are known to occur between the beginning (1 month) and the end (about 6 months) of the chronic phase of the disease, especially regarding the type of immune response which is operating and the consequences of metacestode growth on liver cell metabolism, proliferation and/or death, and on liver fibrosis. Studies in the mouse experimental models are highly relevant to the pathogenesis of AE in humans, since rodents represent *E. multilocularis* intermediate hosts in nature; the identification of transcriptional responses associated with experimental AE may thus provide insight into disease pathogenesis and suggest novel intervention strategies to improve outcome of a still deadly disease in humans.

The aim of this study was to use expression profiling to define transcriptional patterns and regulatory pathways that characterize the host liver response to *E. multilocularis* infection in the experimental model of secondary infection, to compare gene expression in this model to those described in the model of ‘primary infection’, and to follow the changes in gene expression and in the expression of a cell proliferation marker over time during the complete chronic phase of *E. multilocularis* infection, following its 3 stages: initial, middle and late.

## Results

### Animal model and parasitic lesions

For all experiments at each time point, mice were matched for age and weight. After infection, the infected mice had alveolar echinococcosis of the liver as evidenced by the presence of hepatic liver lesions (n = 8/10, 10/10, 10/10, and 10/10 at 1, 2, 3 and 6 months, respectively). Over time, the lesions grew in size and became more extensive and diffuse to the neighborly tissues and organs. Peritoneal metastases appeared at month 2. At month 3, metastases remained localized in the peritoneum, but at month 6, they extended to the lung and diaphragm in 6 of 10 mice, and protoscoleces were present in all parasitic lesions. Individual lesions exhibited the same morphology including a central parasitic vesicle of approximately 1–2 mm to 4–6 mm of diameter, surrounded by a 0.5–1.0 mm-thick white periparasitic inflammatory corona. The average size of liver lesions was 2.5 mm (1–4 mm) at 2 months, 6 mm (2–18 mm) at 3 months, and 20 mm (14–32 mm) at 6 months. Microscopic examination found the typical pathological aspect of *E. multilocularis*-induced lesions (*data not shown*). Sham-infected control mice did not present any macroscopically or microscopically visible lesions in the liver.

### Hepatic injury induced by *E. multilocularis*

Pathological changes in the livers of the mice infected with *E. multilocularis* were observed by microscopy at 1, 2, 3 and 6 months post-infection. One month after infection, fatty degeneration was found in hepatocytes, and fibroblasts proliferated. Lymphocytes were present in portal spaces and Kupffer cells proliferated from 2 months to 6 months. There was no evidence of necrosis or apoptosis in the liver lobules, centro-lobular area and/or portal spaces distant from the parasitic lesions (*data not shown*).

### Distinct transcriptional signatures in the liver of mice during *E. multilocularis* infection

**Figure 1:**
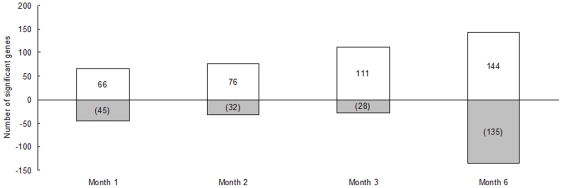
Distribution of genes influenced by *E. multilocularis* infection according to the time of infection. The number of genes significantly up- (positive values) and down-regulated (negative values) is shown at each time point.

Changes of the mouse hepatic gene expression in response to *E. multilocularis* infection in the liver were examined at 1 and 2 months (initial stage of the chronic infection phase), 3 months (middle stage) and 6 months (late stage). Five hundred and fifty-seven differentially expressed genes were found in *E. multilocularis*-infected versus control mice at four time-points with the threshold of 1% false discovery rate. Age-matched *E. multilocularis* infected mice exhibited altered gene expression as defined by Database for Annotation, Visualization and Integrated Discovery (DAVID) software with default settings. A total of 111, 108, 139 and 279 genes were significantly different between control mice and infected mice at 1, 2, 3 and 6 months after infection respectively (FDR adjusted P-value of <0.05). The time course of the respectively up-regulated and down-regulated genes is given in Figure 1. The number of up-regulated genes was higher than that of down-regulated genes in the initial and middle stage of infection; it became nearly equal in the late stage of infection. The number of both up-regulated and down-regulated genes was rather stable in the initial stage of infection, slightly increased in the middle stage, and markedly increased in the late stage.

### Functional analysis of differentially expressed genes

Functional categorization of genes that were differentially expressed between *E. multilocularis*-infected and non-infected mice at each time point (months 1, 2, 3, and 6) after infection was performed using the expression analysis systematic explorer software. There were several biological processes involved over the entire 6 months time period pertaining to an active infection, including gene products associated with the defense response, immune response, acute phase response, antigen presentation and processing, MHC and MHC receptor activity, apoptosis, and cell proliferation, represented in Table S2. Gene ontology (GO; www.geneontology.org) analysis showed that changes were observed among genes involved in response to wounding, response to stress, immune response, defense response, inflammatory response, biosynthetic processes, antigen processing and presentation, and chemokine activity. Additionally, a limited set of antigen presentation-related GO categories, including antigen presentation, antigen processing, major histocompatibility complex (MHC) class I, MHC class II, and MHC class I and II receptor activity, was enriched along the course of infection. Further GO analysis specifically examining gene expression at month 6, when the differential transcriptional activity peaked, showed that genes up-regulated in mice were primarily immunity- and cell proliferation-related, whereas down-regulated genes were associated with catalytic activity and oxidation reduction (Table S1).

Immune response genes, including the acute phase response, MHC, macrophage, T and B cell development, and complement which represented most of the up-regulated genes at 1 month (Table S2), continued to be up-regulated throughout the 6-month experiment. The acute phase lipocalin family and serum amyloid family were prominent members of this group.

### Time course of differential gene expression across the various stages of *E. multilocularis* chronic infection

Multiple ‘biological process ontology clusters’, generated by the microarray analysis, characterized hepatic changes during the time course of the infection by *E. multilocularis* at each stage of its development. They included immune response, pathogen response, and biological processes, as shown in Table S2. After 1 month of infection, top 10 up-regulated genes were involved in defense response, immune response and response to wounding, while top 10 down-regulated genes were involved in metabolism and transport (Table 1). After 2 months of infection, top 10 up-regulated genes were associated with response to stress and biosynthetic process, while top 10 down-regulated genes were involved in transport and cytoskeleton (Table 2). After 3 month of infection, top 10 up-regulated genes were involved in cell proliferation and signal transduction, while top 10 down-regulated genes were involved in metabolism and biosynthetic process (Table 3). After 6 month of infection, top 10 up-regulated genes were associated with inflammatory response and signal transduction, while top 10 down-regulated genes were involved in metabolism and transport (Table 4).

**Table 1:** Top 10 up- or down-regulated differentially expressed genes and their GO clustering classification at 1 month after intrahepatic injection of *E. multilocularis* metacestode (infected mice), compared to intrahepatic injection of saline (non-infected mice).

Change	Gene ID	Gene Symbol	Name	Fold change	Classification
Up-	12655	Chi313	chitinase 3-like 3	137.15	Defense response
	57262	Retnla	resistin like alpha	43.12	Receptor binding
	12642	Ch25h	cholesterol 25-hydroxylase	31.29	Metabolism
	20307	Ccl8	chemokine (C-C motif) ligand 8	29.58	Immune response
	76507	Abp1	amiloride binding protein 1	6.63	Response to stimulus
	21786	Tff3	trefoil factor 3, intestinal	5.91	Secreted
	20293	Ccl12	chemokine (C-C motif) ligand 12	5.64	Immune response
	14038	Expi	extracellular proteinase inhibitor	5.41	Enzyme inhibitor activity
	19222	Ptgir	prostaglandin I receptor	5.13	G-protein coupled receptor protein signaling pathway
	56619	Clec4e	C-type lectin domain family 4, member e	5.08	Immune response
Down-	15495	Hsd3b4	hydroxy-delta-5-steroid dehydrogenase, 3 beta- and steroid delta-isomerase 4	−8.75	Metabolism
	15496	Hsd3b5	hydroxy-delta-5-steroid dehydrogenase, 3 beta- and steroid delta-isomerase 5	−8.60	Metabolism
	57429	Sult5al	sulfotransferase family 5A, member 1	−5.74	Catalytic activity
	20703	Serpinald	serine (or cysteine) peptidase inhibitor, clade A, member 1d	−4.57	Enzyme inhibitor activity
	11816	Apoe	apolipoprotein E	−4.14	Metabolism
	13649	Egfr	epidermal growth factor receptor	−4.13	Signal pathway
	11625	Ahsg	alpha-2-HS-glycoprotein	−4.08	Response to external stimulus
	12266	C3	complement component 3	−3.98	Response to wounding
	17709	COX2	cytochrome c oxidase II, mitochondrial	−3.85	Transport
	99571	Fgg	fibrinogen, gamma polypeptide	−3.43	Response to wounding

**Table 2:** Top 10 up- or down-regulated differentially expressed genes and their GO clustering classification at 2 months after intrahepatic injection of *E.multilocularis* metacestode (infected mice), compared to intrahepatic injection of saline (non-infected mice).

Change	Gene ID	Gene Symbol	Name	Fold change	Classification
Up-	11865	Arntl	aryl hydrocarbon receptor nuclear translocator-like	9.59	Transport
	14104	Fasn	fatty acid synthase	6.22	Metabolism
	12575	Cdkn1a	cyclin-dependent kinase inhibitor 1A (P21)	5.60	Response to stress
	103988	Gck	glucokinase	5.00	Biosynthetic process
	170439	Elovl6	ELOVL family member 6, elongation of long chain fatty acids (yeast)	4.64	Biosynthetic process
	212980	Slc45a3	solute carrier family 45, member 3	4.08	Transport
	74246	Gale	galactose-4-epimerase, UDP	4.03	Metabolism
	23882	Gadd45g	growth arrest and DNA-damage-inducible 45 gamma	3.98	Signal transduction
	22151	Tubb2a	tubulin, beta 2a	3.69	Cytoskeleton
	100102	Pcsk9	proprotein convertase subtilisin/kexin type 9	3.57	Response to stress
Down-	230822	A330049M08Rik	RIKEN cDNA A330049M08 gene	−5.58	Cytoskeleton
	94179	Krt23	keratin 23	−5.03	Cytoskeleton
	13170	Dbp	D site albumin promoter binding protein	−3.57	Metabolism
	12368	Casp6	caspase 6	−3.44	Apoptosis
	13108	Cyp2g1	cytochrome P450, family 2, subfamily g, polypeptide 1	−2.89	Transport
	103844	AI842396	expressed sequence AI842396	−2.82	Oxidation reduction
	14373	G0s2	G0/G1 switch gene 2	−2.72	Cell cycle
	68067	3010026009Rik	RIKEN cDNA 3010026O09 gene	−2.70	Unknown
	105171	Arrdc3	arrestin domain containing 3	−2.67	Intracellular part
	68736	1110034B05Rik	RIKEN cDNA 1110034B05 gene	−2.53	Unknown

**Table 3:** Top 10 up- or down-regulated differentially expressed genes and their GO clustering classification at 3 months after intrahepatic injection of *E.multilocularis* metacestode (infected mice), compared to intrahepatic injection of saline (non-infected mice).

Change	Gene ID	Gene Symbol	Name	Fold change	Classification
Up-	16006	Igfbp1	insulin-like growth factor binding protein 1	6.94	Cell proliferation
	16071	IGK-C	immunoglobulin kappa chain,constant region	6.91	Immune response
	14245	Lpin1	lipin 1	6.85	Metabolism
	331535	Serpina7	serine (or cysteine) peptidase inhibitor, clade A (alpha-1 antiproteinase, antitrypsin), member 7	6.75	Signal transduction
	60599	Trp53inp1	transformation related protein 53 inducible nuclear protein 1	5.41	Response to stress
	23882	Gadd45g	growth arrest and DNA-damage-inducible 45 gamma	4.92	Signal transduction
	13119	Cyp4a14	cytochrome P450, family 4, subfamily a, polypeptide 14	4.50	Transport
	234724	Tat	tyrosine aminotransferase	4.16	Biosynthetic process
	53315	Sult1d1	sulfotransferase family 1D, member 1	4.01	Metabolism
	100702	Mpa2l	macrophage activation 2 like	3.99	Immune response
Down-	27375	Tjp3	tight junction protein 3	−4.09	Protein binding
	13370	Dio1	deiodinase, iodothyronine, type I	−3.28	Biosynthetic process
	18761	Prkcq	protein kinase C, theta	−3.22	Signal transduction
	56695	Pnkd	paroxysmal nonkinesiogenic dyskinesia	−3.22	Hydrolase activity
	98845	Eps8l2	EPS8-like 2	−2.94	Signal transduction
	20384	Sfrs5	splicing factor, arginine/serine-rich 5	−2.82	Metabolism
	101502	Hsd3b7	hydroxy-delta-5-steroid dehydrogenase, 3 beta- and steroid delta-isomerase 7	−2.80	Biosynthetic process
	11808	Apoa4	Apolipoprotein A-IV	−2.75	Metabolism
	69585	Hfe2	hemochromatosis type 2 (juvenile)	−2.66	Lipid binding
	108114	Slc22a7	solute carrier family 22 (organic anion transporter), member 7	−2.64	Transport

**Table 4:** Top 10 up-regulated or down-regulated differentially expressed genes and their GO clustering classification at 6 months after intrahepatic injection of *E.multilocularis* metacestode (infected mice), compared to intrahepatic injection of saline (non-infected mice).

Change	Gene ID	Gene Symbol	Name	Fold change	Classification
Up-	17748	Mt1	metallothionein 1	58.26	Response to stimulus
	16819	Lcn2	lipocalin 2	29.78	Response to stimulus
	17750	Mt2	metallothionein 2	25.51	Response to stimulus
	23882	Gadd45g	growth arrest and DNA-damage-inducible 45 gamma	21.94	Signal transduction
	16006	Igfbp1	insulin-like growth factor binding protein 1	14.22	Cell proliferation
	20208	Saa1	serum amyloid A 1	11.63	Inflammatory response
	20210	Saa3	serum amyloid A 3	9.69	Inflammatory response
	18406	Orm2	orosomucoid 2	8.94	Inflammatory response
	76905	Lrg1	leucine-rich alpha-2-glycoprotein 1	8.64	Cell differentiation
	76574	Mfsd2	major facilitator superfamily domain containing 2	7.76	Transport
Down-	84112	Sucnr1	succinate receptor 1	−11.4	Signal transduction
	57430	Sult3a1	sulfotransferase family 3A, member 1	−7.2	Catalytic activity
	17840	MUP1	major urinary protein 1	−7.01	Transport
	17841	Mup2	major urinary protein 2	−6.73	Transport
	14308	Fshb	follicle stimulating hormone beta	−6.66	Cell proliferation
	15278	Tfb2m	transcription factor B2, mitochondrial	−6.43	Metabolism
	17843	Mup4	major urinary protein 4	−5.68	Transport
	17844	Mup5	major urinary protein 5	−5.04	Transport
	108687	Edem2	ER degradation enhancer, mannosidase alpha-like 2	−4.83	Biosynthetic process
	16601	Cabc1	chaperone, ABC1 activity of bc1 complex like (S. pombe)	−4.72	Metabolism

More precisely, at 1 month post-infection, several biological processes relating to an active infection, as defined by GO cluster classification, were involved, including genes mostly associated with the response to external stimuli, response to wounding, immune response, response to stress, chemokine activity, defense response, MHC-related functions, regulation of metabolism, inflammatory response and GTPase activity (Table S2). At 2 months post-infection, the response to stress, response to external stimulus and regulation of metabolism were maintained, and heat shock protein activity, response to temperature stimulus, regulation of biological processes and response to biotic stimuli were added. At 3 months post-infection, the immune response and regulation of metabolism were maintained; cell proliferation, apoptosis and oxido-reductase activity were added. At 6 months post-infection, the inflammatory response, response to stress, response to external stimuli, response to wounding and regulation of metabolism were maintained, and complement activity, antigen presentation, and antigen processing via MHC class II were added among top 10 classification clustering. In addition, at that final stage of the parasitic disease, new up-regulated genes were mostly signal transduction and cell proliferation genes, and new down-regulated genes were mostly transport, metabolism and biosynthetic process, genes especially cytochrome P450 family genes. Metabolism of xenobiotics, which plays a central role in the detoxification of environmental xenobiotics and drugs, consists of 3 ‘phases’: phase I (Cytochrome P450) includes oxidation of xenobiotics, phase II (Glutathione S-Transferases) deals with the conjugation of phase I products and phase III (ATP-dependent transporter) represents the membrane transport system which eliminates phase II metabolites from cells. Table 5 shows that genes involved in every phase of the metabolism of xenobiotics were significantly modified by *E.multilocularis* infection. Two phase I cytochrome P450 genes (Cyp3a13 and Cyp4a14) were up-regulated, 2 phase II glutathione S-transferases (Gsta3 and Gstt3) were down-regulated and the phase III ATP-binding cassette transporter (Abcd3) was up-regulated. In addition, there were many down-regulated genes involved in these 3 subgroups at any stage of infection (Table 5).

**Table 5:** Genes involved in drug xenobiotic metabolisms altered in the liver by *E. multilocularis* infection.

Metabolism	Gene ID	Gene Symbol	Gene description	Fold change			
				Month 1	Month 2	Month 3	Month 6
Phase I	13089	Cyp2b13	cytochrome P450, family 2, subfamily b, polypeptide 13				−2.62
	13098	Cyp2c39	cytochrome P450, family 2, subfamily c, polypeptide 39				−2.08
	13099	Cyp2c40	cytochrome P450, family 2, subfamily c, polypeptide 40				−2.64
	545288	Cyp2c67	cytochrome P450, family 2, subfamily c, polypeptide 67				−2.08
	433247	Cyp2c68	cytochrome P450, family 2, subfamily c, polypeptide 68				−2.08
	13107	Cyp2f2	cytochrome P450, family 2, subfamily f, polypeptide 2				−2.95
	13108	Cyp2g1	cytochrome P450, family 2, subfamily g, polypeptide 1		−2.89		
	13113	Cyp3a13	cytochrome P450, family 3, subfamily a, polypeptide 13			2.29	
	56388	Cyp3a25	cytochrome P450, family 3, subfamily a, polypeptide 25				−2.27
	337924	Cyp3a44	cytochrome P450, family 3, subfamily a, polypeptide 44				−2.14
	13119	Cyp4a14	cytochrome P450, family 4, subfamily a, polypeptide 14			4.50	7.29
	64385	Cyp4f14	cytochrome P450, family 4, subfamily f, polypeptide 14				−2.25
	13123	Cyp7b1	cytochrome P450, family 7, subfamily b, polypeptide 1				−3.50
II	14859	Gsta3	glutathione S-transferase, alpha 3				−2.07
	103140	Gstt3	glutathione S-transferase, theta 3				−2.00
III	27404	Abca8b	ATP-binding cassette, sub-family A (ABC1), member 8b		−2.26		
	19299	Abcd3	ATP-binding cassette, sub-family D (ALD), member 3		2.61		

### Metabolic pathway analysis

In order to view each individual gene and its relationship with other genes in a comprehensive picture over the 3 stages of infection, we focused on pathways among all available annotation terms, according to the Kyoto Encyclopedia of Genes and Genomes (KEGG) pathways annotation. Pathways that were enriched with up- or down-regulated genes during the three stages (initial, middle and late stage) are listed in Table 6. At the initial stage of *E. multilocularis* infection (month 1–2), most of up-regulated pathways involved were related to immune processes and cell trafficking such as antigen processing and presentation, T cell receptor signaling, chemokine signaling, and gap junction signaling (up-regulated) and complement and coagulation cascades (down-regulated), but also to cell proliferation such as mitogen-activated protein kinase (MAPK) signaling (up-regulated), and to xenobiotic metabolism (down-regulated). At the middle stage of *E. multilocularis* infection (month 3), MAPK signaling pathway was maintained and PPAR signaling pathway emerged. At the late stage of *E. multilocularis* infection (month 6), most of up-regulated pathways involved were related to PPAR signaling pathway, complement and coagulation cascades, antigen processing and presentation pathway, adherens junction and cell adhesion molecules, while down-regulated pathways were related to metabolism of xenobiotics by cytochrome P450, gap junctions and drug metabolism (Table 6).

**Table 6:** Pathways enriched with differentially expressed genes during all three stages of *E. multilocularis* infection in experimental mice: initial (1 and 2 months), middle (3 months) and late (6 months) stages.

Stage	Change	Pathway	*P* Value
Initial	Up	Antigen processing and presentation	4.83E-04
		NOD-like receptor signaling pathway	5.84E-04
		Hematopoietic cell lineage	2.31E-03
		Steroid biosynthesis	1.50E-02
		T cell receptor signaling pathway	4.20E-02
		Cytokine-cytokine receptor interaction	2.28E-02
		Chemokine signaling pathway	5.14E-02
		Gap junction	7.05E-02
		MAPK signaling pathway	7.37E-02
	Down	Complement and coagulation cascades	5.70E-04
		Drug metabolism	2.57E-02
Middle	Up	PPAR signaling pathway	1.84E-02
		MAPK signaling pathway	3.90E-02
		Drug metabolism	4.57E-02
	Down	None	
Late	Up	PPAR signaling pathway	6.64E-06
		Prion diseases	2.75E-04
		Complement and coagulation cascades	5.87E-04
		Adherens junction	5.09E-03
		Antigen processing and presentation	9.59E-03
		Adipocytokine signaling pathway	2.35E-02
		Asthma	3.68E-02
		Cysteine and methionine metabolism	3.68E-02
		Cell adhesion molecules (CAMs)	5.00E-02
	Down	Drug metabolism	1.05E-11
		Metabolism of xenobiotics by cytochrome P450	5.23E-11
		Steroid hormone biosynthesis	1.61E-08
		Retinol metabolism	2.85E-08
		Linoleic acid metabolism	1.35E-03
		Arachidonic acid metabolism	1.77E-03
		Ascorbate and aldarate metabolism	1.07E-02
		Gap junction	1.28E-02
		Pentose and glucuronate interconversions	1.36E-02
		Nitrogen metabolism	2.44E-02
		Porphyrin and chlorophyll metabolism	4.00E-02

### Quantitative real-time RT-PCR (qRT-PCR) validation of microarray data

**Figure 2:**
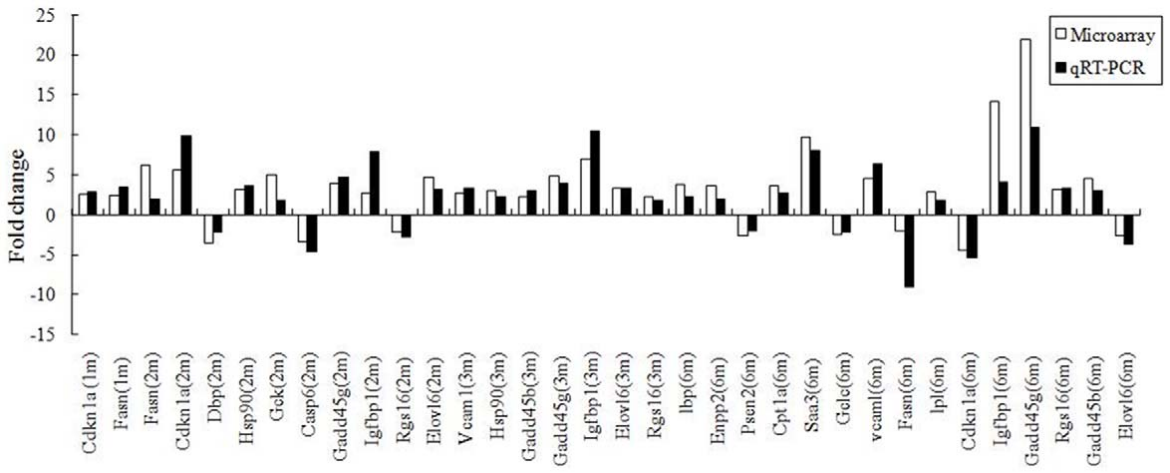
Validation of microarray data by qPCR on 19 randomly selected genes.

Nineteen genes with a differential expression at 2 time-points at least, which were chosen randomly from the four experimental time-points were all confirmed by quantitative real-time RT-PCR. The results from qRT-PCR were highly correlated with those generated from microarray analyses except for Gck at 2 months and Rgs16 at 2 and 3 months (Figure 2).

### Immunohistochemistry of PCNA in liver section

**Figure 3:**
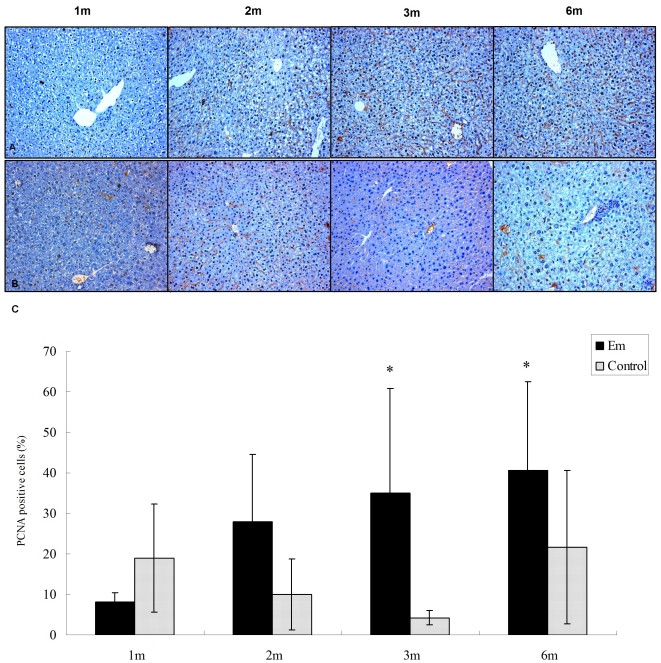
Proliferating Cell Nuclear Antigen (PCNA) expression by hepatic cells in the liver from *E. multilocularis* infected mice (Em) and non-infected mice (control) (histo-immunochemical analysis). A. Hepatic cells close to the parasitic lesions were strongly labeled by the anti-PCNA antibody in *E. multilocularis* infected mice; all cells with a dark-blue/black nucleus are positive cells; some of them indicated by an arrow (initial magnification: ×20). B. Hepatic cells very little expressed PCNA in non-infected mice (initial magnification: ×20). C. Quantitative expression of PCNA was significantly higher in the liver cells of *E. multilocularis* infected mice than in those of non-infected mice at month 3 and 6 (* *p*<0.05).

The expression of PCNA, an important growth marker and DNA replication regulator, was assessed in the liver taken from the *E. multilocularis* infected and uninfected mice. As shown in Figure 3, an increased expression of PCNA was observed in the liver of *E. multilocularis* infected mice compared to the liver of *E. multilocularis* uninfected mice from 2 months to 6 months (Figure 3A and 3B). There was a significant difference between PCNA expression in the hepatocytes of *E. multilocularis* infected and uninfected mice at 3-month and 6-month time-points (*p*<0.05, Figure 3C).

## Discussion

To search for the genes/physiological pathways which characterize *E. multilocularis* influence upon liver parenchyma along the chronic phase of its growth and development, we performed a longitudinal analysis of differentially expressed genes in liver from mice at initial, middle and late stages of the chronic infection phase. *E. multilocularis* infection actually induces a wide range of differential gene expression in the liver and thus a major upheaval in liver growth and metabolism. These deep changes in gene expression increased with time, both for up- and down-regulation, and affected numerous pathways. Functional analysis confirmed that in our model some of the most represented biological process categories were related to the ‘defense response’ and, as already suggested in the primary infection model, were present as early as the 1st month after infection [21]. We also showed that they were sustained all over the chronic stage of the disease. Moreover, in addition to this rather expected finding, we could show that numerous hepatic cell-specific metabolic pathways were disturbed in the liver during the middle and late stages of infection. Some modifications may be related to the inflammatory response, such as the inhibition of a number of structure and transport protein as well as xenobiotic metabolizing enzyme genes. Other modifications indicate a specific influence of the parasite on the hepatocytes, such as cell proliferation and/or apoptosis.

### Genes associated with response to stress and immune/inflammatory response

In *E. multilocularis* infection, the activation of the inflammatory/immune response is obvious in the periparasitic granuloma which characterizes the pathology of AE [12], [22]. However, the diffusion of the gene up-regulation of most of the components of the immune response to the liver parenchyma is a new finding made possible by the microarray technology. It was recently reported in the model of primary infection [21] and is now confirmed in our model of secondary echinococcosis which allows a better separation between a perfectly localized lesion/granuloma and the surrounding liver. It may be ascribed to the immune cell infiltration observed mostly in the portal spaces; however, its intensity suggests that other cells of the liver, hepatocytes, hepatic stellate cells, Kupffer cells, or sinusoidal cells, participate in the observed increased gene expression of most of these components, including chemokines and components of the MHC class I and II-dependent antigen presentation and processing pathways. Chemokines seem to be particularly relevant to the chronic phase of infection and up-regulated in the liver. Chemokine gene up-regulation was also among the most prominent gene changes found at one month in the model of primary AE [21]. In our study, CCL8, CCL12 and CCL17 were up-regulated 30-fold, 6-fold and 3-fold at 1 month, respectively. These chemokines are very potent chemotactic factors for immune cells. CXCL-9, a proinflammatory chemokine, induced by interferon-gamma, and which supports Th1-cell mediated tissue inflammation, was up-regulated 3-fold at 6 months. Observation of such up-regulation of the corresponding genes in the liver itself, in both models of AE, and not only within the periparasitic granuloma, confirm that the surrounding liver is fully involved in a process which was long considered to be a localized “tumor-like” event. Similarly, up-regulation of the genes involved in cell adhesion and leukocyte trans-endothelial migration confirms the involvement of the liver parenchyma in the initiation of vascular neogenesis [9] and its contribution to the constitution and maintenance of the granuloma.

Stress related genes, such as genes which code for Heat Shock Proteins (HSP) 1, 8, 70 and 90, were significantly up-regulated in the liver of infected mice at month 1, 2, 3 and 6. HSPs play a critical role in the recovery of cells from stress and in cytoprotection [22]. HSPs are induced in the host after various types of stress, including infection with parasites, and might be involved as signals to promote and maintain immune tolerance [23]. Therefore, their permanent up-regulated expression could play a critical role in the sustained tolerance against the metacestode. A role for other stress-related proteins, such as MICA/B, has already been suggested to achieve tolerance induction/maintenance in *E. multilocularis* infection [24]. But the role of HSPs has until now been totally underestimated and never studied in this disease, although an HSP70 from parasite origin and close homology with human HSP70 was discovered a few years ago in a cestode of the same genus, *E. granulosus* [25]. Amazingly, up-regulation of HSP genes was not observed at 1 month after primary infection [21], perhaps because this time point corresponds, in fact, to events more precocious in primary than secondary infection and/or because the pathogen- or damage-associated molecular patterns (PAMPs and DAMPs) that induce HSP gene activation are different in primary infection (through *E. multilocularis* oncospheres) and in secondary infection (through *E. multilocularis* metacestode). Inhibition of effector cell functions may also be involved in the tolerance to the parasite. As Protein Kinase C (PKC) is the enzyme responsible for initiation of oxidative mechanisms in macrophages, which play an important role in the development of protective immunity [26], the inhibition of protein kinase C (PKC) gene in the liver of mice infected with *E. multilocularis* at 3 months (Table S2) may suggest one possible mechanism used by *E. multilocularis* to evade the host immune response, as was observed in *Leishmania spp* [26] and *T. congolense* [27] infections.

Lipocalin 2 (Lcn2) was a prominent gene up-regulated in our infected mice at 6 months (almost 30 fold) (Table 4), but was not recognized as such at the initial stage of our study or in primary *E. multilocularis* infection at 1 month [21]. Lcn2, a 25-kDa glycoprotein of the lipocalin superfamily has been associated with the transport of fatty acids and iron [28], [29], the induction of apoptosis [28], [30], and the modulation of inflammatory responses [28]. Lcn2 expression is induced in various cells under harmful conditions such as cancer, infection, and more generally inflammation [28], [31]–[33]. As genes of other members of the lipocalin family, such as major urinary proteins (alpha 2 microglobulins) 1, 2, 3 and 4 were also markedly elevated at 6 months, changes in lipocalins seem to characterize the late stage of *E. multilocularis* infection. After 6 months of *E. multilocularis* infection, the genes of a hallmark inflammation protein in mice, Serum Amyloid A [34], [35], were also markedly up-regulated, up to 12-, 10- and 2 -fold for Saa1, Saa3 and Saa4 respectively. SAA is an apolipoprotein associated with high density lipoproteins, thus also related to lipid metabolism and transport; it is involved in the transport of cholesterol to the liver for its secretion into the bile, the recruitment of immune cells to inflammatory sites, and the induction of enzymes that degrade extracellular matrix. In *E. multilocularis*-infected C57BL/6 mice, amyloidosis, consisting of a mixture of serum amyloid A1 (SAA1) and (SAA2)-derived AA protein was detected in the kidney, liver and spleen of the experimental animals [36]–[38]. An ‘alveolar hydatid cyst-related amyloidosis enhancing factor’ was identified in these mice but not completely characterized and the role of lipid peroxidation in SAA clearance and AA fibril formation was suggested [36]–[38]. A number of other acute phase protein genes were also overexpressed in infected animals, such as orosomucoid and leucin-rich alpha-2-glycoprotein 1 which peaked at 6 months with a 9-fold increase, and also properdin, haptoglobin or hemopexin. However, the significant changes in lipocalins as well as SAAs in the liver of mice induced by *E. multilocularis* infection may be of particular significance and related to the changes in lipid metabolism and up-regulation of adipocytokines found in our study and also pointed out in the model of primary infection [21]. In addition to their other functions as acute phase proteins, Lcn2 and SAAs could also be involved in the development of fibrosis. This might explain their peak at the late phase of the disease. In AE, fibrosis may both limit the parasitic lesion development and be harmful to the liver [12]. Hepatic stellate cells (fat-storing cells/lipocytes) and their morphological/functional changes into extracellular matrix-producing myofibroblasts are crucial in the liver fibrosis process [39]. Electron microscopy of the human liver surrounding *E. multilocularis* lesions [40] and immunostaining of the liver in experimental animals [1] showed abundance of such cells. Their role, together with Lcn2 and SAAs and deep changes in lipid metabolism, should certainly be studied with a renewed approach.

### Genes associated with cell proliferation and death and signal transduction

Liver regeneration is a response to injury, and cell proliferation is essential to restore hepatic function. Although hepatocyte proliferation is often mediated by the injury/regeneration response, in other circumstances it is part of an adaptive response to stress stimuli that are not sufficient to lead to cell death (direct hyperplasia). MAPK signaling is one of the major pathways which regulate the balance between cell survival and cell death in acute and chronic liver injury [41]. Our previous studies suggested that *E. multilocularis* could directly affect hepatocyte proliferation and interact with the MAPK pathway [15]. Microarray profiling confirms and extends the impact of this interaction. In our study, many MAPK family members were up-regulated at the initial and middle stages of *E. multilocularis* infection, including PKC, Gadd45b, Gadd45g, Rap1b, whereas Gadd45a was down-regulated. Growth arrest and DNA damage 45 (GADD45) family genes regulate cell cycle and apoptosis by their direct interaction with critical cell cycle and cell survival regulatory proteins, such as PCNA [42], p21 (WAF1/CIP1), MTK/MEKK4, an upstream activator of the JNK pathway, and Cdc2 protein kinase. Induction of GADD45 expression is involved in the regulation of cell differentiation, cell cycle progression, and apoptosis. In addition, GADD45 family proteins associate with CDK1 (Cdc2-p34) and inhibit the kinase activity of the CDK1-cyclinB1 complex, which mediate the G2/M cell cycle arrest in response to genotoxic stress [43]. Involvement of the GADD45 family proteins has also been implicated in regulating the S-phase checkpoint following UV irradiation and in DNA damage repair. Our present data showed that Gadd45? was up-regulated at month 3 and 6, whereas Gadd45a, a p53-regulated and DNA damage inducible protein, was down-regulated at month 2 in *E. multilocularis* infected mice. Gadd45? is a striking marker of the immediate-early phase of hepatocyte cell proliferation; its action might be to protect hepatocytes from apoptosis, and it is activated by TNF-?, a cytokine known to be expressed at the periphery of the periparasitic granuloma, at the border of the liver parenchyma, in human AE [40], [44]. Promotion of the proliferation of the hepatocytes through this pathway is also confirmed by our observation of an increased expression of PCNA, a subunit of the mammalian DNA polymerase delta synthesized primarily during the S phase of the cell cycle [45] which functions as a molecular integrator for proteins involved in the control of the cell cycle (Figure 3). PCNA is a good marker of proliferating cells and, as mentioned above, binds to Gadd45 molecules. Influence of the parasite on hepatic cell proliferation when the parasitic infection becomes chronic is also supported by the up-regulation of metallothioneins (Mt) 1 and 2, Bcl2, and insulin-like growth factor binding protein 1 (Igfbp1), and by the down-regulation of the cyclin dependent kinase inhibitor 1A (p21). Igbfp1 was highly up-regulated in *E. multilocularis* infected mice at 2, 3 and 6 months. Igfbp1 binds both insulin growth factor (Igf) 1 and Igf2, two major growth factors, with high affinity. Igfbp1 putatively functions as a critical survival factor in the liver by suppressing the level and activation of specific pro-apoptotic factors via its regulation of integrin-mediated signaling [46]. Conversely, as is often observed in microbial attacks, genes of the metabolic pathways involved in apoptosis were also present. Together with Gadd45gamma, an inhibitor of cell growth and apoptosis inducer, which was among the top 10 up-regulated genes at 3 and 6 months, caspase 3 was up-regulated in the liver of mice infected with *E.multilocularis* at 3 months (Table S2). Caspase 3 is a member of the interleukin-1 beta-converting enzyme or cell death effector-3 family, which is involved in the induction of apoptosis and the most prevalent downstream enzyme in their apoptosis-inducing pathway [47], [48]. These observations are consistent with results obtained in other parasitic diseases [49]–[55]. Apoptosis could result from either toxic by-products originating from *E. multilocularis* or from parasite-induced immune cytotoxicity and contribute to the induction of hepatic cell proliferation.

### Genes associated with metabolism and transport

Genes involved in all phases I, II and III of xenobiotic metabolism were altered in mice infected with *E. multilocularis*, especially in the late stage of the disease. As seen in other rodent and human studies of hepatic injury, several members of the cytochrome P450 (CYP) family were differentially expressed during *E. multilocularis* infection in mice. At 6 months, most of the CYPs were down-regulated (Table 5). This finding is in agreement with other studies which showed that expression and activities of cytochrome P450 enzymes were down-regulated in the liver during host response to bacteria [56], [57], *Plasmodium berghei* [58] and *Leishmania donovani* infection [59]. Members of the cytochrome P450 family are major actors in detoxification of xenobiotics, and play key roles in steroid, lipid, and bile acid metabolism. Reactive oxygen species are postulated to contribute to DNA damage and electrophilic cytochrome P450 molecules are a major source of these highly reactive radicals [60]. It has been suggested that decrease in CYP gene expression could be an adaptive or homeostatic response as the liver needs to devote its transcriptional machinery to the synthesis of acute phase proteins that play important roles in controlling the systemic inflammatory response [61], [62]. The glutathione pathway plays a critical role in the detoxification of many drugs and xenobiotics. In this study, we observed that Gsta3 and Gstt3 were decreased about 2-fold after *E. multilocularis* infection at 6 months time-point (Table 5). In addition, there were two ATP binding cassette transporter genes altered (1 up-regulated and 1 down-regulated) (Table 5). These phase III transporters, localized to the cell membrane, also play a role in drug availability, metabolism and toxicity resulting in protection of cells and tissues against xenobiotics. The biological relationship of these genes and *E. multilocularis* infection warrants further investigations and could perhaps explain that hepatic toxicity is more frequent when antiparasitic benzimidazole drugs are used to treat echinococcosis than other diseases [63].

In addition, the expression of a number of genes encoding transporters that were not previously known to be affected by *E. multilocularis* infection is also of interest. Changes in a number of genes of the solute transporter family were observed all along the course of infection and peaked at 6 months. These transporters take part in the absorption and/or reabsorption of carbohydrates, amino acids and metals. Down-regulation of the expression of these genes related to transport may ultimately lead to malnutrition, as is observed in infected mice at the late stage and AE patients with severe disease. Down-regulation of other genes involved in fatty acid and phospholipid metabolism such as acyl-CoA synthetase short-chain family member 2 [64] might contribute to the wasting syndrome commonly observed in the infected mice after 6 months of infection.

In summary, we characterized time-dependent gene expression signatures in the liver of mice infected with *E. multilocularis*. Transcription profiles yield a consistent ranking of differentially expressed genes. They well fit with some of the characteristic features of *E. multilocularis* infection in mice, and especially the important inflammatory and immunological changes previously described using more conventional methods. They also explain some abnormalities observed in patients with AE which had not been well understood before, such as weight loss, hepatomegaly, liver fibrosis or abnormalities in drug metabolism [65]. In addition, they suggest new approaches to study host-parasite relationship and more common events in the liver such as regeneration/cell proliferation or fibrosis.

## Materials and Methods

### Ethics Statement

All animals received humane care in compliance with the Medical Research Center's guidelines, and animal procedures were approved by the Animal Care and Use Committee and the Ethical Committee of First Affiliated Hospital of Xinjiang Medical University (20081205-2).

### Mice and experimental design

Pathogen-free female BALB/c mice (8–10-week old) purchased from animal center of Xinjiang Medical University were housed in cages with a 12-h light/dark cycle and provided with rodent chow and water. *Echinococcus multilocularis* (*E*. *multilocularis*) metacestodes were obtained from intraperitoneal lesions maintained in *Meriones unguiculatus*, and 0.1 mL of pooled lesions (∼1, 000 protoscoleces), was injected into the anterior liver lobe of infected mice as previously described [66]. For each autopsy time-point, to ensure successful infection and availability of at least 3 mice liver per group for the microarray analysis, ten mice were experimentally infected with *E*. *multilocularis* and five mice, used as controls, received an intra-hepatic injection of 0.1 mL of saline in the anterior liver lobe using the same surgical procedure. Mice were killed at month 1, 2, 3 and 6, respectively.

### Tissue sampling and histopathological examination of the parasitic lesions and of the surrounding liver parenchyma

The presence of parasitic lesions was checked in the liver and adjacent organs; the size of the liver lesion(s) and the weight of metastases, if any, were measured. Protoscolex formation in parasitic lesions was examined microscopically. Liver tissue samples were taken close to the parasitic lesions, i.e. 1–2 mm from the macroscopic changes due to the metacestode/granuloma lesion, thus avoiding liver contamination with infiltrating immune cells and parasitic tissue in *E*. *multilocularis* infected mice or were taken from the same (anterior) liver lobe in control mice. Tissue fragments were separated into two parts and either deep-frozen in liquid nitrogen or formalin-fixed and embedded in paraffin. Routine staining using hematoxylin and eosin was used for histopathology studies.

### Detection of proliferating cell nuclear antigen (PCNA) on liver sections

Liver sections from *E*. *multilocularis* infected mice and from control mice (n = 5, including those 3 samples selected for microarray analysis) for each time point, were immunostained with mouse monoclonal antibody against PCNA (dilution 1∶300; Santa Cruz, CA, USA) according to the manufacturer's instructions. PCNA-positive hepatocytes were counted in three random visual fields of 0.95 square mm each, at initial magnification: ×20, for each sample, and the number expressed as the percent of PCNA positive cells to the total number of cells counted. Sections were examined microscopically for specific staining and photographs were taken using a digital image-capture system (Olympus, Tokyo, Japan).

### RNA processing and microarray analysis

Liver tissue samples of each mouse were processed and analyzed separately. Approximately 50 mm^3^-sized liver tissue samples from *E. multilocularis* infected mice (adjacent by 1 mm to the macroscopically visible parasitic lesion) or same size liver tissue samples from control mice were used to extract total RNA using TRIzol reagent (Invitrogen, Gaithersburg, MD, USA).The quality of RNA was confirmed by use of a formaldehyde agarose gel and the concentration of RNA was determined by reading the absorbance at 260/280 mn. After RNA quality control and histopathological evaluation for possible contamination of the liver by lesions/granuloma, RNA extracts from 3 infected and 3 control mice were randomly selected for each time point for microarray analysis. Total RNA was purified with Nucleospin® RNA Clean-up Kit (Macherey-Nagel, Germany) and each purified RNA sample isolated from an individual sample was run on a single microarray. All microarray procedures were done at the ‘microarray core facility’ of Medical Research Center, First affiliated hospital of Xinxiang Medical University. Mouse genome microarrays were purchased from CapitalBio Corporation (Beijing, China); they consisted of 32’256 optimized 70-mer single strand oligonucleotides, representing about 25,000 well-characterized *Mus musculus* genes. Double-stranded cDNAs (containing the T7 RNA polymerase promoter sequence) were synthesized from 2 µg of total RNA using the CbcScript reverse transcriptase with cDNA synthesis system according to the manufacturer's protocol (CapitalBio Corp, China) with the T7 Oligo (dT). cDNA labeled with a fluorescent dye (Cy5 or Cy3-dCTP) was produced by Eberwine's linear RNA amplification method. The Klenow enzyme labeling strategy was adopted after reverse transcription using CbcScript II reverse transcriptase. All procedures for hybridization, and slide and image processing were carried out according to the manufacturer's instructions. The slides were washed, dried, and scanned using a confocal LuxScanTM scanner and the obtained images were then analyzed using LuxScanTM 3.0 software (both from CapitalBio Corp, China). For each array hybridization, sample from control animal or experiment animal was as test (Cy5, red) versus common control (Cy3, green).

### Data analyses and annotation of gene function

For individual channel data extraction, faint spots for which the intensities were below 400 units after background subtraction in both channels (Cy3 and Cy5) were removed. A space- and intensity-dependent normalization based on a LOWESS program was employed. To avoid false positive results, multiple testing corrections were considered. In each experiment, three types of positive controls (Hex, four housekeeping genes, and eight yeast genes) and two types of negative controls (50% DMSO and twelve negative control sequences from the Operon Oligo database) were used. We performed three independent cDNA microarray experiments to obtain more precise data. Initially, data were viewed as a scatter plot of Cy3 vs. Cy5 intensities. Cy3/Cy5 ratios were determined for the individual gene along with various other quality control parameters (e.g., intensity over local background). The bad spots were manually flagged. Flagged spots were not included in subsequent analysis. The fluorescence ratio of individual gene was obtained by averaging the values of total corresponding spots. The duplicate data for one single RNA sample were averaged for each gene.

Normalized and averaged fluorescence ratios of genes were used to calculate the increase and decrease fold of samples derived from experimental animals compared with the fluorescence ratio of the sample derived from control animals. A threshold of 2-fold change in gene expression was used as the cut-off value.

The original microarray data have been uploaded to Gene Expression Omnibus (GEO) website: http://www.ncbi.nlm.nih.gov/geo/index.cgi. All data is MIAME compliant.

### Gene ontology and KEGG analysis

Functional annotation of the differentially expressed genes (classified as ‘biological process ontology clusters’) was obtained from the Gene Ontology Consortium database, based on their respective molecular function, biological process, or cellular component [67]. Functional annotation and clustering of up- or down-regulated genes discovered in the above procedure was carried out by querying database for annotation, visualization and integrated discovery (DAVID) [68]. Simultaneously, pathways that were enriched with up- or down-regulated genes were extracted out in this procedure. A variant of the one-tailed Fisher exact probability test based on the hypergeometric distribution was used to calculate P value.

The biological interpretation of the gene clusters was further completed by Kyoto Encyclopedia of Genes and Genomes (KEGG) pathways annotation [69].

### Quantitative real-time RT-PCR

Nineteen differential expression genes from different categories and time-points were chosen for quantitative real-time RT-PCR analyses. The housekeeping gene beta-actin was chosen as normalizer. The specific primers for these genes were designed using Primer Express Software (TAKARA, Dalian, China) and were listed in Table 7. Samples of RNA extracted from individual mice were used in the quantitative real-time RT-PCR analyses. cDNA was synthesized from 1 µg of RNA in the presence of ribonuclease inhibitor (Promega, Shanghai, China), dNTPs, Oligo(dT) 18 primers, and RevertAid™ M-Mulv reverse transcriptase in a total of 25 µL reaction mix. Quantitative PCR was performed using the SYBR Green program on the iQ5 Real Time PCR system (Bio-Rad, USA). Cycling parameters were 95°C for 1 min and then 40 cycles of 95°C (5 s), 50–62°C (30 s) followed by a melting curve analysis and all cycle threshold values were normalized to the expression of the housekeeping gene beta-actin. qRT-PCR data and microarray data (normalized intensities) were compared by calculating the overall correlation of liver at all time points in both mouse strains for each gene. RNA expression level fold changes were calculated as described by the SYBR Green I protocol.

**Table 7:** Primers and cycling parameters of qRT-PCR verification for microarray data.

Gene	Genbank accession	Primer Sequences	Annealing temperature	Expected Size
beta-actin	NM_007393	F:5′-AACTCCATCATGAAGTGTGA-3′R:5′-ACTCCTGCTTGCTGATCCAC-3′	60.0°C	248 bp
Fasn	NM_007988	F:5′-AGCTTCGGCTGCTGTTGGA-3′R:5′-CGTCTCGGGATCTCTGCTAAGG-3′	60.0°C	146 bp
Cdkn1a	NM_007669	F:5′-CTGTCTTGCACTCTGGTGTCTGA-3′R: 5′-CCAATCTGCGCTTGGAGTGA-3′	60.0°C	121 bp
Dbp	NM_016974	F: 5′-ATCTCGCCCTGTCAAGCATTC-3′R:5′-TGTACCTCCGGCTCCAGTACTTC-3′	50.9°C	159 bp
Hsp90	NM_010480	F:5′-ATCACGAAGCATAACGACGATGAG-3′R:5′-TGCAAGATAACCTTTGTTCCACGA-3′	50.0°C	125 bp
Gck	NM_010292	F: 5′-CAACTGGACCAAGGGCTTCAA-3′R: 5′-TGTGGCCACCGTGTCATTC-3′	62.0°C	133 bp
Casp6	NM_009811	F: 5′-CAAGTGTCAGAGCCTGGTTGGA-3′R: 5′-ACGGGTACGCTGGCTA-3′	62.0°C	78 bp
Vcam1	NM_011693	F:5′-AGCCTCAACGGTACTTTGGATACTG-3′R:5′- GCCCGTAGTGCTGCAAGTGA-3′	60.0°C	124 bp
Lbp	NM_008489	F: 5′- GATGGAGATCGAAGGCTTTGTGA-3′R: 5′- GCAGCATCCCGGTAACCTTG-3′	60.0°C	125 bp
Enpp2	NM_015744	F: 5′- CATCGGCGTCAATCTCTGCTTA-3′R: 5′- GCAGGATCCAGATGTGTTGGTC-3′	60.0°C	118 bp
Psen2	NM_011183	F:5′-CAAGTCTGTGCGTTTCTACACTGAG-3′R: 5′- AGGGTGTTAAGCACGGAGTTGA-3′	60.0°C	111 bp
Cpt1a	NM_013495	F: 5′- GAAGCCTTTGGGTGGATATGTGA-3′R: 5′- ATGGAACTGGTGGCCAATGA-3′	60.0°C	147 bp
Saa3	NM_011315	F: 5′- TGCATCTTGATCCTGGGAGTTG-3′R: 5′- CCGAGCATGGAAGTATTTGTCTGA-3′	60.0°C	144 bp
Gclc	NM_010295	F: 5′- GATGTGGACACCCGATGCAG-3′R: 5′- CAGGATGGTTTGCAATGAACTCTC-3′	60.0°C	115 bp
Lpl	NM_008509	F:5′-TGAACTCTCAACCATCCTGCCTTAG-3′R: 5′- GGCGGAGATGAGTCTCAAATGAA-3′	60.5°C	147 bp
Gadd45b	NM_008655	F: 5′- GAGGCGGCCAAACTGATGA-3′R: 5′-TCGCAGCAGAACGACTGGA-3′	60.0°C	128 bp
Gadd45g	NM_011817	F: 5′- TGGATAACTTGCTGTTCGTGGA-3′R: 5′-CAGCAGAAGTTCGTGCAGTG-3′	60.0°C	122 bp
Igfbp1	NM_008341	F: 5′- TGGAACGCCATCAGCACCTA-3′R: 5′-CATTCTTGTTGCAGTTTGGCAGAT-3′	60.0°C	175 bp
Rgs16	NM_011267	F: 5′- ACGAGTACATCCGCAGCGAAG-3′R: 5′-AGCCACATCGAAGCAACTGGTAG-3′	60.0°C	110 bp
Elovl6	NM_130450	F: 5′- TCAACGAGAACGAAGCCATCC-3′R: 5′-AGTCAGCGACCAGAGCACGA-3′	60.0°C	158 bp

## Supporting Information

Table S1 Functional (Gene Ontology) categories of significantly differentially expressed genes at month 6 after *E. multilocularis* infection.
(0.04 MB DOC)

Table S2 Differentially expressed genes in the liver of mice at 1, 2, 3 and 6 months after *E. multilocularis* infection compared with non-infected mice.
(0.36 MB PDF)
