# Supplementary material for: Time Course of Gene Expression Profiling in the Liver of Experimental Mice Infected with Echinococcus multilocularis
Source: PLoS One. 2011 Jan 19;6(1):e14557. doi: 10.1371/journal.pone.0014557 (PMC3023716; doi:10.1371/journal.pone.0014557)
Supplement: Table S1 — Functional (Gene Ontology) categories of significantly differentially expressed genes at month 6 after E. multilocularis infection. (0.04 MB DOC) [file pone.0014557.s001.doc]

Table S1. Functional (Gene Ontology) categories of significantly differentially expressed genes at month 6 after *E. multilocularis* infection.

| Up-regulated Genes (*E.multilocularis* infected mice *vs* non-infected mice) | P-value |
| --- | --- |
| Acute inflammatory response | 8.71E-15 |
| Response to stimulus | 2.59E-14 |
| Response to stress | 3.67E-12 |
| Defense response | 6.47E-12 |
| Inflammatory response | 1.18E-11 |
| Response to wounding | 1.81E-10 |
| Response to external stimulus | 1.38E-09 |
| Regulation of immune system process | 2.21E-08 |
| Regulation of response to stimulus | 3.12E-08 |
| Adaptive immune response | 7.10E-07 |
| Complement activation | 1.47E-05 |
| Cytolysis | 4.43E-05 |
| Antigen processing and presentation of exogenous antigen | 4.43E-05 |
|  |  |
| Down-regulated Genes (*E.multilocularis* infected mice *vs* non-infected mice) | P-value |
| catalytic activity | 3.85E-15 |
| oxidation reduction | 9.05E-13 |
| oxidoreductase activity | 5.22E-12 |
| steroid biosynthetic process | 7.39E-11 |
| monooxygenase activity | 2.76E-08 |
| lipid biosynthetic process | 1.22E-07 |
| heme binding | 8.84E-07 |
| metabolic process | 1.50E-06 |
| electron carrier activity | 1.42E-05 |
