# Supplementary material for: Time Course of Gene Expression Profiling in the Liver of Experimental Mice Infected with Echinococcus multilocularis
Source: PLoS One. 2011 Jan 19;6(1):e14557. doi: 10.1371/journal.pone.0014557 (PMC3023716; doi:10.1371/journal.pone.0014557)
Supplement: Table S2 — Differentially expressed genes in the liver of mice at 1, 2, 3 and 6 months after E. multilocularis infection compared with non-infected mice. (0.36 MB PDF) [file pone.0014557.s002.pdf]

Table S2. Differentially expressed genes in the liver of mice at 1, 2, 3 and 6 months after *E. multilocularis* infection compared with non-infected mice.

Gene Symbol	Entrez Gene ID	Gene Description	Month 1	Month 2	Month 3	Month 6	Classification
1110034B05Rik	68736	RIKEN cDNA 1110034B05 gene		-2.53			unknown
1110067D22Rik	216551	RIKEN cDNA 1110067D22 gene		3.07			sugar binding
1190002H23Rik	66214	RIKEN cDNA 1190002H23 gene	3.15				cell cycle
1200006F02Rik	71706	RIKEN cDNA 1200006F02 gene				-2.61	integral to membrane
1300013J15Rik	67473	RIKEN cDNA 1300013J15 gene				-2.06	unknown
1810011O10Rik	69068	RIKEN cDNA 1810011O10 gene			2.51		apoptosis
1810015C04Rik	66270	RIKEN cDNA 1810015C04 gene				3.47	membrane
1810055G02Rik	72056	RIKEN cDNA 1810055G02 gene				4.78	extracellular space
2010003K11Rik	69861	RIKEN cDNA 2010003K11 gene			-2.26		unknown
2010305C02Rik	380712	RIKEN cDNA 2010305C02 gene				-3.15	extracellular space
2310007B03Rik	71874	RIKEN cDNA 2310007B03 gene				-2.53	unknown
2310031A18RIK	69627	RIKEN CDNA 2310031A18 gene				-4.28	unknown
2310076L09Rik	66968	RIKEN cDNA 2310076L09 gene				3.59	unknown
2610028AO1Rik	72400	RIKEN cDNA 2610028A01 gene	-2.01				cell cycle
2610528J11Rik	66451	RIKEN cDNA 2610528J11 gene				-2.53	membrane
3010026009Rik	68067	RIKEN cDNA 3010026O09 gene		-2.70			unknown
3110001K24Rik	75698	RIKEN cDNA 3110001K24 gene			3.26		hydrolase activity
4930504E06Rik	75007	RIKEN cDNA 4930504E06 gene			-2.12		unknown
4930515G01Rik	67642	RIKEN cDNA 4930515G01 gene			2.02		unknown
4930528F23Rik	75178	RIKEN cDNA 4930528F23 gene		-2.20			unknown
5830443L24Rik	76074	RIKEN CDNA 5830443L24 gene			2.84		immune response
8430408G22Rik	213393	RIKEN cDNA 8430408G22 gene	-2.14			-2.06	mitochondrion
A330049M08Rik	230822	RIKEN cDNA A330049M08 gene		-5.58			cytoskeleton
AA986860	212439	expressed sequence AA986860	2.37				cytoplasm
Abca8b	27404	ATP-binding cassette, sub-family A (ABC1), member 8b		-2.26			transport

Table S2. Differentially expressed genes in the liver of mice at 1, 2, 3 and 6 months after *E. multilocularis* infection compared with non-infected mice.

Gene Symbol	Entrez Gene ID	Gene Description	Month 1	Month 2	Month 3	Month 6	Classification
Abcd3	19299	ATP-binding cassette, sub-family D (ALD), member 3		2.61			transport
Abp1	76507	amiloride binding protein 1	6.63				Response to stimulus
Acly	104112	ATP citrate lyase		3.37			biosynthetic process
Acsm2	233799	acyl-CoA synthetase medium-chain family member 2	-2.42				metabolism
Acss2	60525	acyl-CoA synthetase short-chain family member 2				-2.51	metabolism
Acta2	11475	actin, alpha 2, smooth muscle, aorta	2.31				metabolism
ACTG1	11465	actin, gamma, cytoplasmic 1				2.17	cell differentiation and development
Actr6	67019	ARP6 actin-related protein 6 homolog (yeast)			2.63		biosynthetic process
Acy3	71670	Aspartoacylase (aminoacylase) 3			-2.03		metabolism
Adck5	268822	aarF domain containing kinase 5		-2.00			metabolism
Adfp	11520	adipose differentiation related protein				2.63	metabolism
Adh4	26876	alcohol dehydrogenase 4 (class II), pi polypeptide				-2.38	alcohol dehydrogenase 4 (class II)
Adra1b	11548	adrenergic receptor, alpha 1b				-2.16	metabolism
Afmid	71562	arylformamidase				-2.18	metabolism
Agxt	11611	alanine-glyoxylate aminotransferase		-2.37			metabolism
Ahcy	269378	S-adenosylhomocysteine hydrolase		2.05			metabolism
Ahsg	11625	alpha-2-HS-glycoprotein	-4.08				Response to external stimulus
AI132487	104910	expressed sequence AI132487				3.59	transport
AI842396	103844	expressed sequence AI842396		-2.82			oxidation reduction
Akp2	11647	alkaline phosphatase 2, liver				2.01	metabolism
Akr1c14	105387	aldo-keto reductase family 1, member C14				-2.14	oxidoreductase activity
Akr1c19	432720	aldo-keto reductase family 1, member C19		2.25		-2.63	oxidation reduction
Alas1	11655	aminolevulinic acid synthase 1				3.12	biosynthetic process
Alb	11657	albumin 1	-3.12				response to external stimulus
Aldh1l1	107747	aldehyde dehydrogenase 1 family, member L1		2.35			biosynthetic process

Table S2. Differentially expressed genes in the liver of mice at 1, 2, 3 and 6 months after *E. multilocularis* infection compared with non-infected mice.

Gene Symbol	Entrez Gene ID	Gene Description	Month 1	Month 2	Month 3	Month 6	Classification
Aldoc	11676	Aldolase 3, C isoform			2.15		metabolism
Alkbh2	231642	alkB, alkylation repair homolog 2 (E. coli)				2.24	response to stress
Alkbh7	66400	alkB, alkylation repair homolog 7 (E. coli)				-2.10	oxidoreductase activity
Ambp	11699	alpha 1 microglobulin/bikunin (Ambp), mRNA [NM_007443]	-2.35				immune response
Amdhd1	71761	amidohydrolase domain containing 1		-2.14			metabolism
Angptl4	57875	angiopoietin-like 4				2.90	response to stress
Anp32a	11737	acidic(leucine-rich) nuclear phosphoprotein 32 family,member a				-2.24	intracellular membrane-bound organelle
Aox3	71724	aldehyde oxidase 3		-2.02			metabolism
Ap3m1	55946	adaptor-related protein complex 3, mu 1 subunit				2.22	transport
Apcs	20219	serum amyloid P-component				2.39	carbohydrate binding
Apoa2	11807	apolipoprotein A-II	-2.82				metabolism
Apoa4	11808	Apolipoprotein A-IV			-2.75		metabolism
Apoc1	11812	apolipoprotein C-I	-3.20				metabolism
Apoe	11816	apolipoprotein E	-4.14				metabolism
Apol7a	75761	RIKEN CDNA 9130022K13 gene				-2.50	unknown
Apol7c	108956	RIKEN CDNA 2210421G13 gene				-3.09	transport
Arhgef19	213649	Rho guanine nucleotide exchange factor (GEF) 19			-2.30		signal transduction
Arhgef3	71704	Rho guanine nucleotide exchange factor (GEF) 3			2.10	2.10	signal transduction
Arl4a	11861	ADP-ribosylation factor-like 4A			2.64		GTPase activity
Arl6ip2	56298	ADP-ribosylation factor-like 6 interacting protein 2			2.05		immune response
Arntl	11865	aryl hydrocarbon receptor nuclear translocator-like		9.59		-2.57	transcription
Arrdc2	70807	Arrestin domain containing 2			2.22		unknown
Arrdc3	105171	arrestin domain containing 3		-2.67			unknown
Atp1b1	11931	ATPase, Na+/K+ transporting, beta 1 polypeptide				2.29	transport
Atp2c2	69047	ATPase, Ca++ transporting, type 2C, member 2				-2.18	transport

Table S2. Differentially expressed genes in the liver of mice at 1, 2, 3 and 6 months after *E. multilocularis* infection compared with non-infected mice.

Gene Symbol	Entrez Gene ID	Gene Description	Month 1	Month 2	Month 3	Month 6	Classification
AU018778	234564	expressed sequence AU018778				-2.25	hydrolase activity
Baiap2l1	66898	BAI1-associated protein 2-like 1			2.62		biosynthetic process
BC012278	235956	cDNA sequence BC012278			2.31		metabolism
BC014805	236149	cDNA sequence BC014805			2.63		transport
BC029214	227622	cDNA sequence BC029214				-2.67	unknown
BC048507	408058	cDNA sequence BC048507		2.12			cytoskeleton
BC057170	236573	cDNA sequence BC057170			3.41		immune response
Bcar3	29815	breast cancer anti-estrogen resistance 3				-2.37	signal transduction
Bcl2a1b	12045	B-cell leukemia/lymphoma 2 related protein A1b	3.01				cell death
Bcl2a1c	12046	B-cell leukemia/lymphoma 2 related protein A1c	3.14				cell death
C1qb	12260	complement component 1, q subcomponent, beta polypeptide				2.12	immune response
C1QC	12262	complement component 1, q subcomponent, C chain				3.58	inflammatory response
C1qg	12279	Complement C1q subcomponent, C chain precursor.				2.18	inflammatory response
C3	12266	complement component 3	-3.98				response to wounding
C4b	625018	complement component 4B		3.09			immuno-response
C730048C13Rik	319800	RIKEN CDNA C730048C13 gene				-2.68	transport
C8a	230558	complement component 8, alpha polypeptide				3.98	inflammatory response
Cabc1	16601	chaperone, ABC1 activity of bc1 complex like (S. pombe)				-4.72	metabolism
Cacybp	12301	Calcyclin binding protein			2.15		metabolism
Capza2	12343	Capping protein (actin filament) muscle Z-line, alpha 2			2.08		metabolism
Car14	23831	Carbonic anhydrase 14			-2.21	-2.02	metabolism
Car3	12350	carbonic anhydrase 3	-3.40			-3.84	metabolism
Car5a	12352	carbonic anhydrase 5a, mitochondrial				-2.06	metabolism
Casp3	12367	Caspase 3			2.42		apoptosis
Casp6	12368	caspase 6		-3.44			apoptosis

Table S2. Differentially expressed genes in the liver of mice at 1, 2, 3 and 6 months after *E. multilocularis* infection compared with non-infected mice.

Gene Symbol	Entrez Gene ID	Gene Description	Month 1	Month 2	Month 3	Month 6	Classification
Cbr2	12409	carbonyl reductase 2	3.26				metabolism
Cbx3	12417	chromobox homolog 3 (Drosophila HP1 gamma)			2.11		metabolism
Cc15	20304	chemokine (C-C motif) ligand 5	2.35				immune response
Ccar1	67500	cell division cycle and apoptosis regulator 1			2.20	2.16	apoptosis
Ccl12	20293	chemokine (C-C motif) ligand 12	5.64				Immune response
Ccl17	20295	chemokine (C-C motif) ligand 17	3.36				response to external stimulus
Ccl8	20307	chemokine (C-C motif) ligand 8	29.58				Immune response
Cd14	12475	CD14 antigen	2.23				response to wounding
Cd163	93671	CD163 antigen				2.56	inflammatory response
Cd3d	12500	CD3 antigen, delta polypeptide	2.14				immune response
Cd52	23833	CD52 antigen				2.48	immune system process
Cd5l	11801	CD51 antigen-like				2.69	cell death
Cd7	12516	CD7 antigen	2.19				immune response
Cd74	16149	CD74 antigen (invariant polypeptide of major histocompatibility complex, class II antigen-associated)				3.13	antigen processing and presentation
Cd8a	12525	CD8 antigen, alpha chain	4.41				immune response
Cd8b1	12526	CD8 antigen, beta chain 1,	3.13				immune response
Cdkn1a	12575	cyclin-dependent kinase inhibitor 1A (P21)	2.54	5.60		4.42	response to stress
Cebpb	12608	CCAAT/enhancer binding protein (C/EBP), beta				3.67	biosynthetic process
Cfhr1	12628	Complement factor H-related 1			2.66		immune response
cfp	18636	complement factor properdin				2.19	inflammatory response
Ch25h	12642	cholesterol 25-hydroxylase	31.29				metabolism
Chi313	12655	chitinase 3-like 3	137.15				Defense response
Chit1	71884	chitinase 1 (chitotriosidase)	2.30				metabolism
Chn1	108699	chimerin (chimaerin) 1		2.12			signal transduction
Cidea	12683	cell death-inducing DNA fragmentation factor, alpha subunit-like effector A	4.09				metabolism

Table S2. Differentially expressed genes in the liver of mice at 1, 2, 3 and 6 months after *E. multilocularis* infection compared with non-infected mice.

Gene Symbol	Entrez Gene ID	Gene Description	Month 1	Month 2	Month 3	Month 6	Classification
Cks2	66197	CDC28 protein kinase regulatory subunit2				-2.77	cell cycle
Cldn14	56173	claudin 14				3.45	cell-cell adhesion
CLEC1B	56760	C-type lectin domain family 1, member b				2.20	signal transduction
Clec4d	17474	C-type lectin domain family 4, member d	3.97				immune response
Clec4e	56619	C-type lectin domain family 4, member e	5.08				immune response
Cnn1	12797	calponin 1	2.17				steroid delta-isomerase activity
Cnn2	12798	calponin 2				2.07	protein binding
Coq10b	67876	Coenzyme Q10 homolog B (S. cerevisiae)			2.38	4.88	intracellular
COX2	17709	cytochrome c oxidase II	-3.85				Transport
Cpt1a	12894	carnitine palmitoyltransferase 1a, liver				3.55	metabolism
Creld2	76737	cysteine-rich with EGF-like domains 2		2.72			cytoplasm
Crls1	66586	cardiolipin synthase 1			2.07		biosynthetic process
Csrp3	13009	cysteine and glycine-rich protein 3		-2.39			metabolism
Ctla2α	13024	cytotoxic t lymphocyte-associated protein 2 α			2.49		hydrolase activity
Cx3crl	13051	chemokine (C-X3-C) receptor 1	-2.00				protein binding
Cxcl12	20315	chemokine (C-X-C motif) ligand 12				-2.21	cell differentiation
Cxcl9	17329	chemokine (C-X-C motif) ligand 9				2.81	inflammatory response
Cyb5b	66427	cytochrome b5 type B				-2.63	electron transport
Cyp2b13	13089	cytochrome P450, family 2, subfamily b, polypeptide 13				-2.62	electron transport
Cyp2c39	13098	cytochrome P450, family 2, subfamily c, polypeptide 39				-2.08	electron transport
Cyp2c40	13099	cytochrome P450, family 2, subfamily c, polypeptide 40				-2.64	electron transport
Cyp2c67	545288	RIKEN CDNA C730004C24 gene				-2.08	electron transport
Cyp2c68	433247	cytochrome P450, family 2, subfamily c, polypeptide 40				-2.08	electron transport
Cyp2f2	13107	cytochrome P450, family 2, subfamily f, polypeptide 2				-2.95	electron transport
Cyp2g1	13108	cytochrome P450, family 2, subfamily g, polypeptide 1		-2.89			electron transport/oxidation reduction

Table S2. Differentially expressed genes in the liver of mice at 1, 2, 3 and 6 months after *E. multilocularis* infection compared with non-infected mice.

Gene Symbol	Entrez Gene ID	Gene Description	Month 1	Month 2	Month 3	Month 6	Classification
Cyp3a13	13113	cytochrome P450, family 3, subfamily a, polypeptide 13			2.29		metabolism
Cyp3a25	56388	cytochrome P450, family 3, subfamily a, polypeptide 25				-2.27	electron transport
Cyp3a44	337924	cytochrome P450, family 3, subfamily a, polypeptide 44				-2.14	electron transport
Cyp4a14	13119	cytochrome P450, family 4, subfamily a, polypeptide 14			4.50	7.29	metabolism
Cyp4f14	64385	cytochrome P450, family 4, subfamily f, polypeptide 14				-2.25	electron transport
Cyp7b1	13123	cytochrome P450, family 7, subfamily b, polypeptide 1				-3.50	electron transport
D14Ertd436e	218978	DNA SEGMENT, CHR 14, ERATO DOI 436, EXPRESSED				2.70	unknown
D14Ertd449e	66039	Uncharacterized protein C10orf57 homolog				-2.31	membrane
D3Ucla1	28146	stress-associated endoplasmic reticulum protein 1			2.03		transport
D4Bwg0951e	52829	Uncharacterized protein C9orf150 homolog				2.35	unknown
D630002G06Rik	236293	RIKEN cDNA D630002G06 gene				-2.17	transport
Dbp	13170	D site albumin promoter binding protein		-3.57			metabolism
Dcun1d1	114893	DCUN1D1 DCN1, defective in cullin neddylation 1, domain containing 1 (S. cerevisiae)			2.05		unknown
Ddc	13195	dopa decarboxylase		2.17		-4.42	biosynthetic process
Ddx3x	13205	DEAD/H (Asp-Glu-Ala-Asp/His) box polypeptide 3, X-linked			2.92		hydrolase activity
Dgat2	67800	diacylglycerol O-acyltransferase 2		2.15			metabolism
Dhcr24	74754	24-dehydrocholesterol reductase		2.95			response to stress
Dhrs1	52585	dehydrogenase/reductase (SDR family) member 1				-2.01	metabolism
Dio1	13370	deiodinase, iodothyronine, type I	-2.43		-3.28		biosynthetic process
Dnaja1	15502	DnaJ (Hsp40) homolog, subfamily A, member 1		2.24	3.01		response to stress
Dnajb10	56812	DnaJ (Hsp40) homolog, subfamily B, member 10		2.12			metabolism
Dnajb4	67035	DnaJ (Hsp40) homolog, subfamily B, member 4			3.55		metabolism
Dnajb9	27362	DnaJ (Hsp40) homolog, subfamily B, member 9			3.78		protein folding
Dnalc4	54152	dynein, axonemal, light chain 4				-2.07	microtubule-based process
Dscr1	54720	Down syndrome critical region homolog 1 (human)				4.32	cell development

Table S2. Differentially expressed genes in the liver of mice at 1, 2, 3 and 6 months after *E. multilocularis* infection compared with non-infected mice.

Gene Symbol	Entrez Gene ID	Gene Description	Month 1	Month 2	Month 3	Month 6	Classification
Dst	13518	dystonin				2.09	transport
Dusp1	19252	dual specificity phosphatase 1			2.73		cell cycle
Ear11	93726	eosinophil-associated, ribonuclease A family, member 11	4.88				hydrolase activity
Ecgf1	72962	endothelial cell growth factor 1 (platelet-derived)			-2.18		metabolism
Ecm1	13601	extracellular matrix protein 1				-2.26	transport
Edem2	108687	ER degradation enhancer, mannosidase alpha-like 2				-4.83	biosynthetic process
EG13909	13909	predicted gene, EG13909	-3.16				catalytic activity
EG193330	100039226	similar to eukaryotic translation initiation factor 1a,X-chromosomal			2.05		unknown
EG236844	236844	similar to ribosomal protein L22 like 1			2.13		unknown
EG241041	634856	predicted gene, EG241041				-2.29	unknown
EG434674	434674	predicted gene, EG434674				-2.68	unknown
EG624219	624219	predicted gene, EG624219	2.19		-2.15		extracellular region
Egfr	13649	epidermal growth factor receptor (Egfr), transcript variant 2	-4.13			5.75	cell proliferation
Eif1a	13664	eukaryotic translation initiation factor 1A			2.41		biosynthetic process
Eif4ebp3	108112	eukaryotic translation initiation factor 4E binding protein 3				2.26	biosynthetic process
Ell2	192657	elongation factor RNA polymerase II 2			3.17		metabolism
Elmo3	234683	engulfment and cell motility 3, ced-12 homolog		-2.02			apoptosis
Elov13	12686	elongation of very long chain fatty acids (FEN1/Elo2, SUR4/Elo3, yeast)-like 3	-2.67				metabolism
Elovl6	170439	ELOVL family member 6, elongation of long chain fatty acids (yeast)		4.64	3.36	-2.63	biosynthetic process
Eno1	103324	enolase 1, alpha non-neuron		2.11			metabolism
Enpp2	18606	ectonucleotide pyrophosphatase/phosphodiesterase 2				3.61	response to stress
Eps8l2	98845	EPS8-like 2			-2.94		signal transduction
Es1	13884	esterase 1	-2.25				metabolism
Es31	382053	esterase 31				-2.96	carboxylesterase activity
Esrp2	77411	epithelial splicing regulatory protein 2			-2.55		nucleotide binding

Table S2. Differentially expressed genes in the liver of mice at 1, 2, 3 and 6 months after *E. multilocularis* infection compared with non-infected mice.

Gene Symbol	Entrez Gene ID	Gene Description	Month 1	Month 2	Month 3	Month 6	Classification
Ethe1	66071	ethylmalonic encephalopathy 1				-2.52	cytoplasmic part
Etnk2	214253	ethanolamine kinase 2				2.59	transport
Ets2	23872	E26 avian leukemia oncogene2, 3' domain				3.31	metabolism
Expi	14038	extracellular proteinase inhibitor	5.41				enzyme inhibitor activity
F13a1	74145	coagulation factor XIII, A1 subunit	4.89				response to wounding
Fabp1	14080	fatty acid binding protein 1	-2.61				transport
Fabp4	11770	fatty acid binding protein 4, adipocyte			2.06	2.17	metabolism
Fabp5	16592	fatty acid binding protein 5, epidermal			2.40		biosynthetic process
Fahd1	68636	fumarylacetoacetate hydrolase domain containing 1				-2.56	metabolism
Fasn	14104	fatty acid synthase		6.22		-2.09	biosynthetic process
Fbp1	14121	fructose bisphosphatase 1	-2.80				metabolism
Fbxo31	76454	F-box protein 31				2.88	unknown
Fcgr3	14131	Fc receptor, IgG, low affinity III				2.73	inflammatory response
Fdft1	14137	farnesyl diphosphate farnesyl transferase 1				-2.52	metabolism
Ffar2	233079	free fatty acid receptor 2	2.15				G-protein coupled receptor protein signaling pathway
Fgb	110135	fibrinogen, B beta polypeptide	-2.40				response to wounding
Fgf1	14164	fibroblast growth factor 1		2.04			cell cycle
Fgg	99571	fibrinogen, gamma polypeptide	-3.43				response to wounding
Fgl1	234199	fibrinogen-like protein 1			2.28	2.75	signal transduction
Fkbp4	14228	FK506 binding protein 4		2.23			metabolism
Fmo1	14261	flavin containing monooxygenase 1				-2.96	electron transport
Fmo2	55990	flavin containing monooxygenase 2			2.20		metabolism
Fmo3	14262	flavin containing monooxygenase 3		2.07		-3.27	metabolism/oxidation reduction
Fshb	14308	follicle stimulating hormone beta				-6.66	cell proliferation
Gabarapl1	57436	gamma-aminobutyric acid (GABA(A)) receptor-associated protein-like 1		-2.05			cytoplasm

Table S2. Differentially expressed genes in the liver of mice at 1, 2, 3 and 6 months after *E. multilocularis* infection compared with non-infected mice.

Gene Symbol	Entrez Gene ID	Gene Description	Month 1	Month 2	Month 3	Month 6	Classification
Gadd45a	13197	growth arrest and DNA-damage-inducible 45 alpha		-2.03			cell cycle
Gadd45b	17873	growth arrest and DNA-damage-inducible 45 beta			2.19	4.49	apoptosis
Gadd45g	23882	growth arrest and DNA-damage-inducible 45 gamma		3.98	4.92	21.94	signal transduction
Gal3st2	381334	galactose-3-O-sulfotransferase 2				-2.63	biosynthetic process
Gale	74246	galactose-4-epimerase, UDP		4.03		-2.03	metabolism
Galm	319625	galactose mutarotase				-2.32	metabolism
Gas6	14456	growth arrest specific 6		2.36			cell cycle
Gbp3	55932	guanylate nucleotide binding protein 3			2.15		immune response
Gck	103988	glucokinase		5.00			biosynthetic process
Gclc	14629	glutamate-cysteine ligase, catalytic subunit				-2.47	response to stress
Ggh	14590	gamma-glutamyl hydrolase			2.49		metabolism
Ggt6	71522	gamma-glutamyltransferase 6		2.11			metabolism
Gm1381	384198	gene model 1381, (NCBI)		2.03			unknown
GNL3	30877	guanine nucleotide binding protein-like 3 (nucleolar)				-2.50	cell proliferation
GOs2	14373	G0/G1 switch gene 2	2.25	-2.72			cell cycle
Gpbp1	73274	GC-rich promoter binding protein 1			2.02		metabolism
Gpd1	14555	glycerol-3-phosphate dehydrogenase 1 (soluble)			-2.02		biosynthetic process
Gpr171	229323	G protein-coupled receptor 171	2.38				G-protein coupled receptor protein signaling pathway
Gpxl	14775	glutathione peroxidase 1	-2.29				response to stress
Grrp1	72690	PREDICTED: glycine	2.51				complement activation, classical pathway
Gsta3	14859	glutathione S-transferase, alpha 3				-2.07	metabolism
Gstt3	103140	glutathione S-transferase, theta 3				-2.00	metabolism
H2-Aa	14960	histocompatibility 2, class II antigen A alpha				3.41	antigen processing and presentation
H2-Ab1	14961	histocompatibility 2, class II antigen A beta1				2.66	antigen processing and presentation
H2-Ea	14968	histocompatibility 2, class II antigen E alpha				2.77	antigen processing and presentation

Table S2. Differentially expressed genes in the liver of mice at 1, 2, 3 and 6 months after *E. multilocularis* infection compared with non-infected mice.

Gene Symbol	Entrez Gene ID	Gene Description	Month 1	Month 2	Month 3	Month 6	Classification
H2-Eb1	14969	histocompatibility 2, class II antigen E beta				3.15	antigen processing and presentation
Hacl1	56794	2-hydroxyacyl-CoA lyase 1				-2.85	metabolism
Hagh	14651	hydroxyacyl glutathione hydrolase				-2.05	hydrolase activity
Hao3	56185	hydroxyacid oxidase (glycolate oxidase) 3				-2.23	metabolism
Hba-a1	15122	hemoglobin alpha, adult chain 1				2.62	transport
Hbb-b1	15129	hemoglobin, beta adult major chain				3.20	transport
Hc	15139	hemolytic complement				2.03	inflammatory response
Hck	15162	hemopoietic cell kinase				2.14	metabolism
Hdhd3	72748	haloacid dehalogenase-like hydrolase domain containing 3			-2.52	-3.41	metabolism
Hectd2	226098	HECT domain containing 2		-2.02			metabolism
Herpud1	64209	homocysteine-inducible, endoplasmic reticulum stress-inducible, ubiquitin-like domain member 1			2.14	2.37	metabolism
Hes6	55927	hairy and enhancer of split 6 (Drosophila)		2.37		-2.05	metabolism
Hfe2	69585	hemochromatosis type 2 (juvenile) (human homolog)			-2.66		lipid binding
Hhex	15242	hematopoietically expressed homeobox				-3.00	metabolism
Hist1h1c	50708	histone cluster 1, H1c		2.23		-2.44	metabolism
Hmgcs1	208715	3-hydroxy-3-methylglutaryl-Coenzyme A synthase 1			2.19		biosynthetic process
Hmox1	15368	heme oxygenase (decycling) 1				2.29	metabolism
Hnmt	140483	histamine N-methyltransferase			2.62		intracellular
Hp	15439	haptoglobin				2.75	inflammatory response
Hpxn	15458	hemopexin				2.28	transport
Hsd17b2	15486	hydroxysteroid (17-beta) dehydrogenase 2				-2.34	metabolism
Hsd3b1	15492	hydroxy-delta-5-steroid dehydrogenase, 3 beta- and steroid delta-isomerase 1				-2.31	metabolism
Hsd3b3	15494	hydroxy-delta-5-steroid dehydrogenase, 3 beta- and steroid delta-isomerase 3				-3.37	metabolism
Hsd3b4	15495	hydroxy-delta-5-steroid dehydrogenase, 3 beta- and steroid delta-isomerase 4 (Hsd3b4), mRNA [NM_008294]	-8.75				metabolism
Hsd3b5	15496	hydroxy-delta-5-steroid dehydrogenase, 3 beta- and steroid delta-isomerase 5 (Hsd3b5), mRNA [NM_008295]	-8.60				metabolism

Table S2. Differentially expressed genes in the liver of mice at 1, 2, 3 and 6 months after *E. multilocularis* infection compared with non-infected mice.

Gene Symbol	Entrez Gene ID	Gene Description	Month 1	Month 2	Month 3	Month 6	Classification
Hsd3b6	15497	hydroxy-delta-5-steroid dehydrogenase, 3 beta- and steroid delta-isomerase 6				-2.21	metabolism
Hsd3b7	101502	hydroxy-delta-5-steroid dehydrogenase, 3 beta- and steroid delta-isomerase 7		-2.21	-2.80	-2.14	biosynthetic process
Hsp25-ps1	15508	heat shock protein25,pseudogene1		2.28			unknown
Hsp90aa1	15519	heat shock protein 90kDa alpha (cytosolic), class A member 1		3.20	3.03		response to stress
Hsp90ab1	15516	heat shock protein 90kDa alpha (cytosolic), class B member 1		2.41			response to stress
Hspa1a	193740	Heat shock protein 1A	2.38				response to stress
Hspa5	14828	heat shock 70kD protein 5 (glucose-regulated protein)		2.03			response to stress
Hspa8	15481	heat shock protein 8		2.83	2.43		response to stress
Hspb1	15507	heat shock protein 1		3.55			response to stress
Hspb8	80888	heat shock protein 8				2.58	response to stress
I11r1	17082	interleukin 1 receptor-like 1	2.92				immune response
I17r	16197	interleukin 7 receptor	2.25				immune response
Idh1	15926	isocitrate dehydrogenase 1 (NADP+), soluble			2.17		metabolism
Ifi202b	26388	interferon activated gene 202			2.88		immune response
Ifi203	15950	interferon activated gene 203			2.13		immune response
Ifi204	15951	interferon activated gene 204			2.47	2.41	immune response
Ifrd1	15982	interferon-related developmental regulator 1			2.20	2.03	cell differentiation
Igf1	16000	insulin-like growth factor 1	-3.33			-2.00	biosynthetic process
Igfals	16005	insulin-like growth factor binding protein, acid labile subunit				-2.02	cell adhesion
Igfbp1	16006	insulin-like growth factor binding protein 1		2.72	6.94	14.22	cell cycle
Igfbp4	16010	insulin-like growth factor binding protein 4		2.13			immuno-response/cell growth
IGK-C	16071	immunoglobulin kappa chain,constant region			6.91		unknown
Iigp1	60440	interferon inducible GTPase 1			2.11		GTPase activity
Il13ra1	16164	interleukin 13 receptor, alpha 1			2.39		signal transduction
Il17r	16172	interleukin 17 receptor D				2.90	signal transduction

Table S2. Differentially expressed genes in the liver of mice at 1, 2, 3 and 6 months after *E. multilocularis* infection compared with non-infected mice.

Gene Symbol	Entrez Gene ID	Gene Description	Month 1	Month 2	Month 3	Month 6	Classification
Ing4	28019	inhibitor of growth family, member 4			-2.08		apoptosis
Insig1	231070	insulin induced gene 1		2.24		-3.45	response to stress
Itih3	16426	inter-alpha trypsin inhibitor, heavy chain 3				2.98	metabolism
Junb	16477	Jun-B oncogene	2.58				cell cycle
Keg1	64697	kidney expressed gene 1				-3.31	cell cycle
Klf13	50794	Kruppel-like factor 13				2.71	metabolism
Klf3	16599	Kruppel-like factor 3 (basic)				2.81	metabolism
Krt23	94179	keratin 23		-5.03			cytoskeleton
Krtap16-10	71369	keratin associated protein 16-10	-3.29				intracellular part
L3mbt12	214669	l(3)mbt-like 2	-2.02				biosynthetic process
Lat	16797	linker for activation of T cells	2.12				immune response
Lbp	16803	lipopolysaccharide binding protein				3.73	defense response
Lcn2	16819	lipocalin 2				29.78	Response to stimulus
Lect1	16840	leukocyte cell derived chemotaxin 1			3.85	-2.30	signal transduction
Lgals1	16852	lectin, galactose binding, soluble 1				2.02	cell development
Lgals3	16854	lectin, galactose binding, soluble 3	3.24			4.11	carbohydrate binding
Lgmn	19141	legumain				2.35	metabolism
Limd2	67803	LIM domain containing 2		-2.05			zinc ion binding
Lpin1	14245	lipin 1			6.85	7.66	metabolism
Lpl	16956	lipoprotein lipase				2.79	metabolism
Lrfn3	233067	leucine rich repeat and fibronectin type III domain containing 3		2.58			protein binding
Lrg1	76905	leucine-rich alpha-2-glycoprotein 1				8.64	Cell differentiation
Lrrc24	378937	leucine rich repeat containing 24	2.81				signal transduction
Lrrc28	67867	leucine rich repeat containing 28				2.57	protein binding
Ltf	17002	lactotransferrin	3.17				transport

Table S2. Differentially expressed genes in the liver of mice at 1, 2, 3 and 6 months after *E. multilocularis* infection compared with non-infected mice.

Gene Symbol	Entrez Gene ID	Gene Description	Month 1	Month 2	Month 3	Month 6	Classification
Lum	17022	lumican			2.06		protein binding
Ly6a	110454	lymphocyte antigen 6 complex, locus A				2.46	defense response
Ly6d	17068	lymphocyte antigen 6 complex, locus D	4.42				extracellular space
Lyzs	17105	lysozyme				2.38	defense response
Lzp-s	17110	P lysozyme structural			2.14	2.50	defense response
Mad2l2	71890	MAD2 mitotic arrest deficient-like 2 (yeast)			-2.23		cell cycle
Mafb	16658	v-maf musculoaponeurotic fibrosarcoma oncogene family, protein B (avian)				3.22	cell differentiation
Mal2	105853	mal, T-cell differentiation protein 2		2.53			protein binding
Marcks	17118	myristoylated alanine rich protein kinase C substrate			2.09		calmodulin binding
Mat1a	11720	methionine adenosyltransferase I, alpha				2.12	metabolism
Mbd1	17190	methyl-CpG binding domain protein 1				3.80	metabolism
Mcart1	230125	mitochondrial carrier triple repeat 1				2.08	transport
Mefv	54483	Mediterranean fever	2.69				response to wounding
Mfsd2	76574	major facilitator superfamily domain containing 2		2.56	2.50	7.76	transport
Mg11	17312	macrophage galactose N-acetyl-galactosamine specific lectin 1	2.56				carbohydrate binding
Mg12	216864	macrophage galactose N-acetyl-galactosamine specific lectin 2	4.64				carbohydrate binding
Midn	59090	midnolin				3.23	metabolism
Mir16	56209	membrane interacting protein of RGS16				2.17	metabolism
Mmp3	17392	matrix metallopeptidase 3	3.34				metabolism
Mmp7	17393	matrix metallopeptidase 7	4.05				metabolism
Mmp9	17395	matrix metallopeptidase 9	2.74				immune response
Mocs2	17434	molybdenum cofactor synthesis 2				-2.01	biosynthetic process
Mon1a	72825	MON1 homolog A (yeast)			-2.07		transport
Morf4l2	56397	mortality factor 4 like 2		2.01			cell cycle
Mpa2l	100702	macrophage activation 2 like		-2.33	3.99		GTP binding

Table S2. Differentially expressed genes in the liver of mice at 1, 2, 3 and 6 months after *E. multilocularis* infection compared with non-infected mice.

Gene Symbol	Entrez Gene ID	Gene Description	Month 1	Month 2	Month 3	Month 6	Classification
Mpeg1	17476	macrophage expressed gene1				2.38	signal transduction
Mrps22	64655	mitochondrial ribosomal protein S22				-2.03	structural molecule activity
Msr1	20288	macrophage scavenger receptor 1			2.11		transport
Msr2	80891	macrophage scavenger receptor 2		2.25			signal transduction
Mt1	17748	metallothionein 1				58.26	Response to stimulus
Mt2	17750	metallothionein 2				25.51	Response to stimulus
MUP1	17840	major urinary protein 1				-7.01	transport
Mup2	17841	major urinary protein 2				-6.73	transport
Mup4	17843	major urinary protein 4				-5.68	transport
Mup5	17844	major urinary protein 5				-5.04	transport
Nat3	17962	N-acetyltransferase 3	-2.23				metabolism
Nat8b	434057	similar to putative N-acetyltransferase camello1				-2.25	unknown
Ncf1	17969	neutrophil cytosolic factor 1	2.26				response to wounding
Ndrg4	234593	N-myc downstream regulated gene 4	2.04				development
Nfil3	18030	nuclear factor, interleukin 3, regulated		2.76			metabolism
Nnmt	18113	nicotinamide N-methyltransferase		2.73		3.40	cytoplasm
Npc1	18145	Niemann Pick type C1				2.13	metabolism
Npr2	230103	natriuretic peptide receptor 2			-2.39		biosynthetic process
Nr0b2	23957	nuclear receptor subfamily 0, group B, member 2				-2.18	metabolism
Nrg4	83961	neuregulin 4			3.88		cell growth
Nrn1	68404	neuritin 1				-2.53	cell differentiation
Nsbp1	50887	nucleosome binding protein1			2.50		metabolism
Nsdhl	18194	NAD(P) dependent steroid dehydrogenase-like		2.12			biosynthetic process
Nsg1	18196	neuron specific gene family member 1	-3.41				G-protein coupled receptor protein signaling pathway
Nsmce1	67711	non-SMC element 1 homolog (S. cerevisiae)		-2.11			response to stress

Table S2. Differentially expressed genes in the liver of mice at 1, 2, 3 and 6 months after *E. multilocularis* infection compared with non-infected mice.

Gene Symbol	Entrez Gene ID	Gene Description	Month 1	Month 2	Month 3	Month 6	Classification
Nudt7	67528	nudix (nucleoside diphosphate linked moiety X)-type motif 7				-2.78	metabolism
odc1	18263	ornithine decarboxylase, structural 1		2.20			biosynthetic process
OLFML1	244198	olfactomedin-like1				-2.34	unknown
Olfr1215	258451	olfactory receptor 1215	-2.53				G-protein coupled receptor protein signaling pathway
Olfr2	18317	olfactory receptor 2		2.49	3.24		signal transduction
Oprs1	18391	opioid receptor, sigma 1				-2.47	metabolism
Orm1	18405	orosomucoid 1				2.61	inflammatory response
Orm2	18406	orosomucoid 2		2.67		8.94	immuno-response/response to stress
Osbp13	71720	oxysterol binding protein-like 3	2.02				metabolism
Osgin1	71839	oxidative stress induced growth inhibitor 1				-2.13	cell growth
OTTMUSG00000016571	433520	similar to zinc finger protein 97			2.66		nucleic acid binding
Pbx1	18514	pre B-cell leukemia transcription factor 1			2.19		immune response
Pcdh1	75599	protocadherin 1			-2.25		metabolism
Pck1	18534	phosphoenolpyruvate carboxykinase 1, cytosolic				2.43	metabolism
Pcsk9	100102	proprotein convertase subtilisin/kexin type 9		3.57		-2.05	response to stress
Pde9a	18585	phosphodiesterase 9A			-2.29	-2.47	signal transduction
Pdlim3	53318	PDZ and LIM domain 3	2.58				protein binding
Pex11a	18631	peroxisomal biogenesis factor 11a				2.06	peroxisome organization and biogenesis
Pglyrp1	21946	peptidoglycan recognition protein 1	3.30				response to external stimulus
Picalm	233489	phosphatidylinositol binding clathrin assembly protein			2.01		immune response
Pira3	18726	paired-Ig-like receptor A3				2.33	signal transducer activity
Pla2g7	27226	phospholipase A2, group VII (platelet-activating factor acetylhydrolase, plasma)				2.57	inflammatory response
Pltp	18830	phospholipid transfer protein				2.08	transport
Pmvk	68603	phosphomevalonate kinase				-2.50	metabolism
Pnkd	56695	paroxysmal nonkinesiogenic dyskinesia			-3.22	-2.20	hydrolase activity

Table S2. Differentially expressed genes in the liver of mice at 1, 2, 3 and 6 months after *E. multilocularis* infection compared with non-infected mice.

Gene Symbol	Entrez Gene ID	Gene Description	Month 1	Month 2	Month 3	Month 6	Classification
Pnrc1	108767	proline-rich nuclear receptor coactivator 1		2.12			receptor activity
Por	18984	P450 (cytochrome) oxidoreductase				3.23	electron transport
Ppara	19013	peroxisome proliferator activated peceptor alpha				2.17	metabolism
Ppfibp2	19024	protein tyrosine phosphatase, receptor-type, F interacting protein, binding protein 2			-2.25		metabolism
Ppp1r14c	76142	protein phosphatase 1, regulatory (inhibitor) subunit 14c	-3.16				enzyme inhibitor activity
Ppp1r3c	53412	protein phosphatase 1, regulatory (inhibitor) subunit 3C				-4.06	transport
Ppp2r5e	26932	protein phosphatase 2, regulatory subunit B (B56), epsilon isoform				2.05	signal transduction
Pqlc2	212555	PQ loop repeat containing 2		-2.06			unknown
Prepl	213760	prolyl endopeptidase-like				2.71	metabolism
Prg4	96875	proteoglycan 4 (megakaryocyte stimulating factor, articular superficial zone protein)				2.52	extracellular space
Prkcq	18761	protein kinase C, theta			-3.22		Signal transduction
Prnd	26434	prion protein dublet		2.42	3.78		metabolism
Pros1	19128	protein S (alpha)				2.29	response to stress
Prss8	76560	protease, serine, 8 (prostasin)			2.14		metabolism
Psen2	19165	presenilin 2				-2.59	cell death
Pter	19212	phosphotriesterase related				-2.13	metabolism
Ptgds	19215	prostaglandin D2 synthase				-3.11	metabolism
Ptgir	19222	prostaglandin I receptor (IP)	5.13				G-protein coupled receptor protein signaling pathway
Ptp4a1	19243	protein tyrosine phosphatase 4a1				2.07	cell migration
Ptp4a1	627166	protein tyrosine phosphatase 4a1			2.08		cell cycle
Ptpn1	19246	protein tyrosine phosphatase, non-receptor type 1				2.32	signal transduction
Ptpn7	320139	protein tyrosine phosphatase, non-receptor type 7	2.65				cytoplasm
Ptprz1	19283	protein tyrosine phosphatase, receptor type Z, polypeptide 1	-2.86				metabolism
Qdpr	110391	quinoid dihydropteridine reductase				-2.06	biosynthetic process
Rab11fip1	75767	RAB11 family interacting protein 1 (class I)				-2.29	transport

Table S2. Differentially expressed genes in the liver of mice at 1, 2, 3 and 6 months after *E. multilocularis* infection compared with non-infected mice.

Gene Symbol	Entrez Gene ID	Gene Description	Month 1	Month 2	Month 3	Month 6	Classification
Rac2	19354	RAS-related C3 botulinum substrate 2				2.29	signal transduction
Ranbp2	19386	RAN binding protein 2			2.16		metabolism
Rap1b	215449	RAS related protein 1b			2.05		signal transduction
Rasgef1b	320292	gpi-gamma4				3.48	signal transduction
Rbm3	19652	RNA binding motif protein 3			3.40		response to temperature stimulus
Rbp1	19659	retinol binding protein 1, cellular				2.37	metabolism
Rbp4	19662	retinol binding protein 4, plasma	-2.44				transport
Rdh9	103142	retinol dehydrogenase 9				2.81	metabolism
Retnla	57262	resistin like alpha	43.12				receptor binding
Rgs16	19734	regulator of G-protein signaling 16		-2.13	2.25	3.23	signal transduction
Rgs18	64214	regulator of G-protein signaling 18	2.02				G-protein coupled receptor protein signaling pathway
Rik3r5	320207	phosphoinositide-3-kinase, regulatory subunit 5, p101	2.20				catalytic activity
Rnf125	67664	ring finger protein 125				-2.81	metabolism
Rnf13	24017	ring finger protein 13			2.13		metabolism
RP23-34B24.1	230161	similar to bile acid coenzyme A:aminoacid N-acyltransferase		2.10			metabolism
Rpl30	19946	ribosomal protein L30			2.01		biosynthetic process
Rpl5	19983	ribosomal protein L5			2.01		biosynthetic process
Rplp1	56040	ribosomal protein, large, P1	-2.08				biosynthetic process
Rxrg	20183	retinoid X receptor gamma				-2.02	transcription
S100a4	20198	S100 calcium binding protein A4	3.88				protein binding
Saa1	20208	serum amyloid A 1				11.63	inflammatory response
Saa3	20210	serum amyloid A 3				9.69	inflammatory response
Saa4	20211	serum amyloid A 4				2.32	inflammatory response
Samhd1	56045	SAM domain and HD domain, 1				2.16	signal transduction
Sat1	20229	spermidine/spermine N1-acetyl transferase 1				2.40	metabolism

Table S2. Differentially expressed genes in the liver of mice at 1, 2, 3 and 6 months after *E. multilocularis* infection compared with non-infected mice.

Gene Symbol	Entrez Gene ID	Gene Description	Month 1	Month 2	Month 3	Month 6	Classification
Sc5d	235293	sterol-C5-desaturase (fungal ERG3, delta-5-desaturase) homolog (S. cerevisae)				-3.33	metabolism
Scd1	20249	stearoyl-Coenzyme A desaturase 1		3.54	2.35		biosynthetic process
Sdc1	20969	syndecan 1		2.18			cytoskeleton
Sdc3	20970	syndecan 3		2.67			cytoskeleton
Sdpr	20324	serum deprivation response			2.65		protein binding
Sdro	70061	orphan short chain dehydrogenase/reductase				-2.39	metabolism
Sds	231691	serine dehydratase				3.82	metabolism
Selenbp1	20341	selenium binding protein 1				-2.37	transport
Sema3g	218877	cDNA fis, clone TRACH3033868, highly similar to Homo sapiens semaphorin sem2	2.74				cell differentiation
Senp1	223870	SUMO1/sentrin specific peptidase 1	-2.52				hydrolase activity
Sepp1	20363	selenoprotein P, plasma, 1	-2.62				metabolism
Serpina3h	546546	serine (or cysteine) peptidase inhibitor, clade A, member 3H				2.89	hydrolase activity
Serpina3k	20714	serine (or cysteine) peptidase inhibitor, clade A, member 3K	-3.38				immune response
Serpina3n	20716	serine (or cysteine) peptidase inhibitor, clade A, member 3N				3.12	inflammatory response
Serpina7	331535	serine (or cysteine) peptidase inhibitor, clade A (alpha-1 antiproteinase, antitrypsin), member 7			6.75	3.49	enzyme regulator activity
Serpinald	20703	serine (or cysteine) peptidase inhibitor, clade A, member 1d	-4.57				enzyme inhibitor activity
Serpinc1	11905	serine (or cysteine) peptidase inhibitor, clade C (antithrombin), member 1	-2.21				response to wounding
Setd4	224440	SET domain containing 4		-2.25			intracellular part
Sfrs10	20462	splicing factor, arginine/serine-rich 10			2.72		metabolism
Sfrs2	20382	splicing factor, arginine/serine-rich 2 (SC-35)		2.15			metabolism
Sfrs5	20384	splicing factor, arginine/serine-rich 5 (SRp40, HRS)			-2.82		metabolism
Sh3bgrl	56726	sh3-binding domain glutamic acid-rich protein like			2.04		nucleus
Shmt1	20425	serine hydroxymethyl transferase 1 (soluble)				-2.97	metabolism
Slc10a1	20493	solute carrier family 10 (sodium/bile acid cotransporter family), member 1				-2.12	transport
Slc12a1	20495	solute carrier family 12, member 1				-3.84	transport

Table S2. Differentially expressed genes in the liver of mice at 1, 2, 3 and 6 months after *E. multilocularis* infection compared with non-infected mice.

Gene Symbol	Entrez Gene ID	Gene Description	Month 1	Month 2	Month 3	Month 6	Classification
Slc22a7	108114	solute carrier family 22 (organic anion transporter), member 7			-2.64		transport
Slc25a22	68267	solute carrier family 25 (mitochondrial carrier, glutamate), member 22				3.12	transport
Slc25a25	227731	solute carrier family 25 (mitochondrial carrier, phosphate carrier), member 25		2.06		2.05	transport
Slc2a6	227659	solute carrier family 2 (facilitated glucose transporter), member 6	2.00				transport
Slc3a1	20532	solute carrier family 3, member 1				3.30	transport
Slc45a3	212980	solute carrier family 45, member 3		4.08			transport
Slpi	20568	secretory leukocyte peptidase inhibitor		3.12			hydrolase activity
Socs3	12702	suppressor of cytokine signaling 3				3.97	metabolism
Spbc24	67629	spindle pole body component 24 homolog (S. cerevisiae)				-2.60	cell cycle
Sponl	233744	spondin 1, (f-spondin) extracellular matrix protein	2.15				protein binding
Spp1	20750	secreted phosphoprotein 1			3.54		immune response
Sqle	20775	squalene epoxidase		2.29			metabolism
Srebf1	20787	sterol regulatory element binding factor 1				-2.51	response to stress
Srgn	19073	serglycin	3.40		2.63	2.34	apoptosis
Srp54	24067	signal recognition particle 54			2.52		GTP binding
Srp9	27058	signal recognition particle 9			2.06		biosynthetic process
St3gal5	20454	ST3 beta-galactoside alpha-2,3-sialyltransferase 5				5.62	metabolism
Stab1	192187	stabilin 1				2.04	inflammatory response
Steap4	117167	STEAP family member 4		2.75	2.66	6.08	oxidation reduction
Stip1	20867	stress-induced phosphoprotein 1		2.72			response to stress
Stra6	20897	stimulated by retinoic acid gene 6	-2.03				transport
Strn3	94186	striatin, calmodulin binding protein 3			2.28		cell cycle
Sucnr1	84112	succinate receptor 1				-11.40	signal transduction
Sult1c2	69083	sulfotransferase family, cytosolic, 1C, member 2		-2.39			metabolism
Sult1d1	53315	sulfotransferase family 1D, member 1			4.01		metabolism

Table S2. Differentially expressed genes in the liver of mice at 1, 2, 3 and 6 months after *E. multilocularis* infection compared with non-infected mice.

Gene Symbol	Entrez Gene ID	Gene Description	Month 1	Month 2	Month 3	Month 6	Classification
Sult3a1	57430	sulfotransferase family 3A, member 1				-7.20	catalytic activity
Sult5al	57429	sulfotransferase family 5A, member 1	-5.74				catalytic activity
Synj2bp	24071	synaptojanin 2 binding protein			2.28		transport
Tardbp	230908	TAR DNA binding protein			2.03		metabolism
Tat	234724	tyrosine aminotransferase		2.38	4.16	2.05	biosynthetic process
Tfb2m	15278	transcription factor B2, mitochondrial				-6.43	metabolism
Tff3	21786	trefoil factor 3, intestinal	5.91				Secreted
Tgfb1i4	21807	TSC22 domain family,member1				-3.20	metabolism
Tgtp	21822	T-cell specific GTPase				2.79	inflammatory response
Thra	21833	thyroid hormone receptor alpha		-2.44			metabolism
Thrsp	21835	thyroid hormone responsive SPOT14 homolog (Rattus)			2.29		nucleus
Tieg1	21847	kruppel-like factor 10				3.06	cell differentiation
Tjp3	27375	tight junction protein 3			-4.09	-2.11	protein binding
Tk1	21877	thymidine kinase 1				-2.14	metabolism
Tmed5	73130	transmembrane emp24 protein transport domain containing 5			2.08	2.32	transport
Tmem150	232086	transmembrane protein 150		2.00		-2.44	protein binding
Tmem176b	65963	transmembrane protein 176B				2.13	cell differentiation
Tnfaip8l1	66443	tumor necrosis factor, alpha-induced protein 8-like 1		-2.14			unknown
Tnmt	21743	indolethylamine N-methyltransferase				-2.54	methyltransferase activity
Tppp3	67971	RIKEN cDNA 2700055K07 gene	2.47				unknown
Trf	22041	transferrin	-2.27				transport
Trp53inp1	60599	transformation related protein 53 inducible nuclear protein 1			5.41	3.38	apoptosis
Tsc22d3	14605	TSC22 domain family 3		2.18	2.20		apoptosis
Tspan4	64540	tetraspanin 4		2.59			cytoplasm
Ttc23	67009	tetratricopeptide repeat domain 23				2.07	binding

Table S2. Differentially expressed genes in the liver of mice at 1, 2, 3 and 6 months after *E. multilocularis* infection compared with non-infected mice.

Gene Symbol	Entrez Gene ID	Gene Description	Month 1	Month 2	Month 3	Month 6	Classification
Tuba2	22143	tubulin, alpha 2		2.06		-2.40	cytoskeleton
Tuba4a	22145	tubulin, alpha 4				-2.32	transport
Tuba6	22146	tubulin, alpha 6		2.76		-2.56	cytoskeleton
Tubb2a	22151	tubulin, beta 2a		3.69		-2.29	cytoskeleton
Tubb2c	227613	tubulin, beta 2c		2.60			cytoskeleton
Tubb2c-ps1	100042651	tubulin, beta 2c, pseudogene 1				-2.64	transport
Ubd	24108	ubiquitin D	3.07			2.26	metabolism
Ube3a	22215	ubiquitin protein ligase E3A			2.10		metabolism
Ucp2	22228	uncoupling protein 2 (mitochondrial, proton carrier)				2.98	transport
UGT1A10	394430	UDP glucuronosyltransferase 1 family, polypeptide A10				-2.90	metabolism
Ugt1a5	394433	udp glucuronosyltransferase1 family,polypeptide a5			2.52	-2.59	metabolism
Ugt2a3	72094	UDP glucuronosyltransferase 2 family, polypeptide A3				-2.48	metabolism
Ugt2b1	71773	UDP glucuronosyltransferase 2 family, polypeptide B1				-2.30	metabolism
Ugt2b37	112417	UDP glucuronosyltransferase 2 family, polypeptide B37				-2.46	metabolism
Ulk1	22241	Unc-51 like kinase 1 (C. elegans)				2.96	cell development
Unc84b	223697	unc-84 homolog B (C. elegans)				2.45	membrane
Upp2	76654	uridine phosphorylase 2			2.81	2.42	metabolism
Vbp1	22327	von Hippel-Lindau binding protein 1			2.12		protein folding
Vcam1	22329	vascular cell adhesion molecule 1			2.67	4.54	protein binding
Vill	22349	villin 1	2.68				metabolism
Vim	22352	vimentin		2.21			cytoplasm
Wasl	73178	Wiskott-Aldrich syndrome-like (human)				2.09	metabolism
Wdr33	74320	WD repeat domain 33				-2.02	transport
Wdr81	192652	WD repeat domain 81			-2.16		metabolism
Wfdc1	67866	WAP four-disulfide core domain 1				-2.01	defense response

Table S2. Differentially expressed genes in the liver of mice at 1, 2, 3 and 6 months after *E. multilocularis* infection compared with non-infected mice.

Gene Symbol	Entrez Gene ID	Gene Description	Month 1	Month 2	Month 3	Month 6	Classification
Wwp1	107568	WW domain containing E3 ubiquitin protein ligase 1			2.37		immune response
Ypel3	66090	yippee-like 3 (Drosophila)		-2.00			unknown
Ywhag	22628	3-monooxygenase/tryptophan 5-monooxygenase activation protein, gamma polypeptide		2.32			developmental process
Zbp1	58203	Z-DNA binding protein 1			2.05	2.23	hydrolase activity
Zc3h6	78751	zinc finger CCCH type containing 6	-2.48				hemopoiesis
Zfp36l1	12192	zinc finger protein 36, C3H type-like 1		2.09			biosynthetic process
Zfp97	22759	zinc finger protein 97			2.32		metabolism
